# Wild eel microbiome reveals that skin mucus of fish could be a natural niche for aquatic mucosal pathogen evolution

**DOI:** 10.1186/s40168-017-0376-1

**Published:** 2017-12-21

**Authors:** Miguel Carda-Diéguez, Rohit Ghai, Francisco Rodríguez-Valera, Carmen Amaro

**Affiliations:** 1 Department of Microbiology and Ecology abd Estructura de Recerca Interdisciplinar en Biotecnologia i Biomedicina (ERI BIOTECMED), University of Valencia, Valencia, Spain; 2 Institute of Hydrobiology, Department of Aquatic Microbial Ecology, Biology Center of the Academy of Sciences of the Czech Republic, České Budějovice, Czech Republic; 3 Evolutionary Genomics Group, Department de Producción Vegetal y Microbiología, Universidad Miguel Hernández, San Juan de Alicante, Spain

**Keywords:** *Vibrio*, Skin mucus, Microbiome, Metagenomics, Attached microbiota

## Abstract

**Background:**

Fish skin mucosal surfaces (SMS) are quite similar in composition and function to some mammalian MS and, in consequence, could constitute an adequate niche for the evolution of mucosal aquatic pathogens in natural environments. We aimed to test this hypothesis by searching for metagenomic and genomic evidences in the SMS-microbiome of a model fish species (*Anguilla Anguilla* or eel), from different ecosystems (four natural environments of different water salinity and one eel farm) as well as the water microbiome (W-microbiome) surrounding the host.

**Results:**

Remarkably, potentially pathogenic *Vibrio* monopolized wild eel SMS-microbiome from natural ecosystems, *Vibrio anguillarum/Vibrio vulnificus* and *Vibrio cholerae/Vibrio metoecu*s being the most abundant ones in SMS from estuary and lake, respectively. Functions encoded in the SMS-microbiome differed significantly from those in the W-microbiome and allowed us to predict that successful mucus colonizers should have specific genes for (i) attachment (mainly by forming biofilms), (ii) bacterial competence and communication, and (iii) resistance to mucosal innate immunity, predators (amoeba), and heavy metals/drugs. In addition, we found several mobile genetic elements (mainly integrative conjugative elements) as well as a series of evidences suggesting that bacteria exchange DNA in SMS. Further, we isolated and sequenced a *V. metoecus* strain from SMS. This isolate shares pathogenicity islands with *V. cholerae* O1 from intestinal infections that are absent in the rest of sequenced *V. metoecus* strains, all of them from water and extra-intestinal infections.

**Conclusions:**

We have obtained metagenomic and genomic evidence in favor of the hypothesis on the role of fish mucosal surfaces as a specialized habitat selecting microbes capable of colonizing and persisting on other comparable mucosal surfaces, e.g., the human intestine.

**Electronic supplementary material:**

The online version of this article (10.1186/s40168-017-0376-1) contains supplementary material, which is available to authorized users.

## Background

Accidental pathogens are bacteria that live in the environment and occasionally infect humans. In the particular case of the aquatic environment, accidental pathogens usually present a life cycle in which they shift between free and sessile life styles, the latter by forming biofilms on abiotic or biotic surfaces. Free-living aquatic pathogens use active motility to swim towards nutrients (positive chemotaxis) that are more abundant on water/surface interfaces. Once on the surface, they may lose motility, attach to, multiply, and colonize the surface by forming biofilms.

One of the most nutrient-rich surfaces available to aquatic pathogens is mucosal surfaces of fish, in particular, the skin mucous surface (SMS). It has been suggested before that teleost SMS share many characteristics with type I mucosal surfaces of mammalian intestines, respiratory tract, and uterus [1]. Similar to these type I mucosal surfaces, teleost SMS is also formed by mucus-secreting cells arranged with epithelial cells and a diffuse skin-associated lymphoid-tissue containing macrophages, mast cells, dendritic cells, and lymphocytes [2, 3]. Furthermore, fish mucosal secretions also contain a wide variety of innate immune molecules including complement proteins, lysozyme, proteases, esterases, lactoferrin (an iron chelator), and anti-microbial peptides [2, 3].

Most studies performed in aquatic accidental pathogens have been focused on disease mechanisms or environmental survival. For instance, in the case of *Vibrio cholerae*, most environmental isolates have been demonstrated to be non-pathogenic, as they lack specific virulence genes located in mobile genetic elements (MGE), mainly phages and pathogenicity islands [4, 5]. Consequently, it has been proposed that pathogenic clones responsible for cholera epidemics have emerged by acquisition of such virulence genes by horizontal gene transfer (HGT) events, either in the natural environment or in the human gut [6].

We hypothesize that the fish SMS could be one of the natural niches for aquatic mucosal pathogen evolution. Accordingly, fish SMS could act as an intermediate niche between water and human mucus (i.e., intestinal) pre-selecting bacteria best adapted to survive and multiply in mucus and favoring mucus-fitness by DNA exchange. As a first approach to test this hypothesis, we selected the European eel (henceforth “eel”) and sequenced the SMS-metagenome from eels captured in different ecosystems, four natural (two estuaries, one lake, and a river) and one artificial (an intensive eel farm) located close to the Mediterranean Sea or the Atlantic coast of Spain as well as the metagenome of the eel-surrounding water (W-metagenome) from one of the natural ecosystems [7]. We selected eels as the best fish candidate because (i) they lack macroscopic scales and are surrounded by a thick layer of mucus similar in function to mammal mucus [8], which can be easily sampled without significant manipulation; (ii) wild eel SMS can contain accidental or opportunistic human pathogens like *Vibrio vulnificus* [9]; and (iii) eels are eurihaline teleostei that inhabit multiple natural environments (lakes, oceans, ponds…) along its life cycle, thus being in contact with a great variety of microorganisms [7]. In fact, adult eels spawn in the Sargasso Sea, migrate as young larvae towards Europe with the Gulf Stream, arriving 1 to 3 years later. Subsequently, they undergo three metamorphoses while colonizing ponds, lagoons, or lakes before reaching sexual maturity and, finally, migrate back to the Sargasso Sea (more than 6000 km against the Gulf Stream) to spawn, closing their life cycle [7]. In addition, the European eel is a species of commercial interest that is cultured (or grown since reproduction in captivity has not been achieved) in intensive farms. Interestingly, a preliminary analysis of the eel-SMS metagenomes suggested that the superficial mucus harbors a microbiota that is quite distinct from that of the surrounding environment [8]. In that work, no analysis of these metagenomes in terms of composition in bacterial species as well as evaluation of potential pathogenicity or functionality of the microbiome was performed (i.e., distinction between potentially virulent vs non-virulent bacteria).

Taken all together, the main objective of this work was to analyze in depth these metagenomic data under the hypothesis that it might be possible (1) to detect genomic features of microbes best adapted to survive and multiply in mucus, (2) examine if potentially pathogenic genomic elements are detectable in fish SMS vs natural environment, and (3) assess the potential for horizontal gene exchange events in the evolution of aquatic mucosal pathogens (i.e., intestinal human pathogens). In the process, we also sought to obtain a deeper insight into the natural SMS-microbiome that travels with eels in natural and artificial ecosystems.

## Methods

### Sampling and DNA isolation

The sampled habitats, their description, and positional coordinates as well as the main physico-chemical parameters of the water samples are presented in Additional file 1: Figure S1 and Table 1. Nets were strategically placed at different locations in the Nature Parks and trapped wild eels were recovered after 24 h (Additional file 1: Figure S2). We also sampled 20 eels (around 250 g) grown in an intensive eel farm (farmed eels) close to Prat de Cabanes-Torreblanca as well as about 10,000 wild eels (glass eels; average weight per individual, 0.33 g) from various rivers of the Atlantic coast of Spain (Galicia) that were fished and transported to the same farm. Wild and farmed eels were deposited in fishbowls of 100 L (6 or 1000 individuals per bowl, depending on the animal size) containing 10–50 mL of sterile PBS for 20 min, and the detached mucus was collected in sterile glass bottles that were stored at 4 °C (Additional file 1: Figure S2). Wild and farmed eels were returned without damage to their habitats or their respective tanks. In parallel to wild eel sampling in the Ebro Delta, water samples were taken directly with sterile glass bottles and were stored at 4 °C. Water- and mucus bottles were transported to the laboratory and were sequentially filtered through five, 1- and 0.22-μm-pore-size filters by using a peristaltic pump. Finally, the prokaryotic biomass recovered was treated with 1 mg/mL lysozyme and 0.2 mg/mL proteinase K (final concentrations). Nucleic acids were extracted with phenol/chloroform/isoamyl alcohol and chloroform/isoamyl alcohol, and DNA integrity was checked by agarose gel electrophoresis [9].

**Table 1:** Sampling points, types of samples, and designation as well as main physico-chemical parameters of water of the sampled environments

Sampling point^a^	Sample type	Sample designation^b^	SRA accession	Salinity (g/l)	pH	T (°C)
Fish farm close to the Mediterranean sea	Skin mucus from eels	FE_4_^5.3^	SRX702744	4	5.3	24
Rivers (North Atlantic coast: Galicia)		WE_≤1_^7^	SRX702748	≤ 1^c^	7	7
Natural Park: Albufera Lake		WE_1_^9.5^	SRX703656	1	9.5	19
Natural Park: Prado Cabanes		WE_7_^8^	SRX703655	7	8	20
Natural Park: Ebro Delta		WE_3_^8^	SRX702749	3	8	19
		WE_10_^8^	SRX710670	10	8	20
	Water	WE_10_^8^W	SRX710703	10	8	20

^a^Coordinates of each sampling point are indicated in Additional file 2: Table S1

^b^Super- and sub-indexes indicate pH and salinity values, respectively

^c^The salinity of the rivers where eels were fished was ≤ 1 g/l

### Sequencing and assembly

DNA samples were sequenced either by using a FLX sequencer (454 Roche) with Titanium chemistry (Centro Superior de Investigación en Salud Pública [CSISP, Valencia, Spain]) or an Illumina HiSeq 2000 sequencer with pair-end technology (Macrogen [Seoul, Korea]). To this end, 500 bp and 100 pair-end libraries were prepared for Roche and Illumina, respectively. Default values of dynamictrim were run (phred cutoff 13) to quality filter reads [10]. Assembly of 454 metagenomic reads was performed using Geneious Pro 5.4 with a minimum overlap of 50 nucleotides, 95% identity, and allowing 1% of mismatches per read, while Illumina reads were assembled using Velvet (k-mer 51) [11]. All the metagenomes have been previously deposited in NCBI SRA with the following accession codes: SRX702744, SRX702748, SRX702749, SRX703649, SRX703655, SRX703656, SRX710670, and SRX710703 [8, 12]. Only assembled contigs bigger than 1 kb were considered for the following analysis.

### Sequence analysis and annotation of the assembled contigs

The %GC of the metagenomes was determined using the “geecee” program from EMBOSS package [13]. BioEdit software was used to manipulate the sequences [14]. The assembled contigs were annotated using Prodigal [11, 15] and the MG-RAST pipeline [16]. The KEGG and SEED databases were used to analyze metabolic pathways and functional classification of the proteins [17]. To allow the interactive visualization of genomic fragment comparisons, Artemis Comparison Tool ACTv.8 [18] was used. Annotation was refined manually using HHpred [19].

### Community structure using all reads and ribosomal RNA

For taxonomy, the entire datasets were compared using BLASTX or BLASTP [20] from the NCBI NR database (cut off expectation e-value 10^−5^, minimum length 50 bp, and minimum similarity of 95%) and analyzed using MEGAN with the “Percent Identity Filter” active [21]. Ribosomal RNA (rRNA) genes were identified by comparing the data sets against the RDP database [22]. All reads that matched an rRNA sequence with an identity 95% and an alignment length of 100 bases against either the RDP or the LSU database were extracted. The best hit with a taxonomic affiliation was considered a reasonable closest attempt to classify the rRNA sequences.

### Functional classification of reads in SEED categories

In order to categorize the reads obtained, we used the pipeline from MG-RAST. This program automatically annotates reads and then classifies them in SEED categories. We downloaded the results in .xls format and compared abundance using Microsoft Office Excel manually.

### *Vibrio* isolation, identification, and genome sequencing

A volume of 1 mL of water or mucus from each one of the samples was inoculated into 4 mL of the *Vibrio* enrichment medium, Alkaline Peptone Water (APA; 1% peptone extract supplemented with 1% [wt/vol] NaCl at pH 8.6) and incubated for 12 h at 28 °C with agitation (150 rpm). Then, volumes of 0.1 mL of a 1:10,000 dilution were spread on plates containing the *Vibrio* selective media, TCBS (thiosulfate-citrate-bile salts-sucrose) (Conda 1074), and VVM (*Vibrio vulnificus* medium) [23] agar and on the general medium TSA-1 (trypticase soy agar supplemented with 1% [wt/vol] NaCl). Plates were incubated 24 h at 28 °C. Suspected colonies were purified on TSA-1, isolated and lyophilized at − 80 °C in LB-1 (Luria Bertani broth1% [wt/vol] NaCl) supplemented with 20% (vol/vol) glycerol. The isolates were phenotypically identified with API 20E kit (bioMerieux) according to manufacturer’s instructions. Bacterial suspensions in PBS were used as inocula. Examination of the strips was conducted after 24 h using the API Database https://apiweb.biomerieux.com/. In parallel, genomic DNA was extracted from suspected colonies and amplified using PCR targeting rDNA by using universal primers 699R (5′- RGGGTTGCGCTCGTT-3′) and 616V (5′- AGAGTTTGATYMTGGCTCAG-3′) to identify bacteria. The amplicons were sequenced and identified in the Genomic section of the SCSIE (Servicios Centrales de Soporte a la Investigación Experimental) from the Universidad of Valencia (Spain). To identify suspected *V. vulnificus*, primers vvhA-F (5′- CGCCACCCACTTTCGGGCC-3′) and vvhA-R (5′-CC GCGGTACAG GTTGGCGC-3′) were used to amplify the hemolysin gene corresponding exclusively to *V. vulnificus* [24]*.*

Selected strains were sequenced using Illumina HiSeq2000 (Macrogen [Seoul, Korea]), genomic DNA was extracted using the Wizard Genomic DNA extraction kit (Promega), and assembly and annotation was followed as described before.

The contigs assembled from sequencing the strain M12v were compared with the deposited *Vibrio metoecus* and *Vibrio cholerae* O1 El Tor N16961 using GCviewer Comparison tool [25]. Default values were used for BLAST atlas comparison.

### Mobile genetic elements detection

Multiple approaches were used to detect MGE in contigs > 10 kb: on one hand to detect intra-contig variations in GC content, taxonomical annotation, and hexanucleotide usage pattern (HUP) and on the other to look for MGE-signature genes (sgMGE) by using BLASTP. BLASTN and ISfinder and ISbrowser were used to identify plasmids and pathogenic islands deposited in databases [26, 27]. We considered as sgMGE: integrases, transposases, conjugative elements, and phage or viral proteins. HUP values were calculated by using *compseq* program from EMBOSS package [13]. Microsoft Excel tools helped us differentiate genes annotated to the same taxon and visualize the GC variation in the contigs. The workflow diagram is shown in Additional file 1: Figure S3. Contigs that contained genes for ribosomal proteins or for lipopolysaccharide biosynthesis and clustered with contigs identified as putative MGE (cpMGE) but did not contain sgMGE were considered false positives. Intra-contig changes in the taxonomic annotation plus presence of an integrase located next to tRNA or several sgMGE were considered to establish the presence of a MGE in a cpMGE.

Three metagenomes generated in the present work (WE_3_^8^, WE_10_^8^, and WE_10_^8^W), and six metagenomes from various origins that were downloaded from EBI metagenomics or MG-RAST were tested for the protocol (Additional file 1: Table S1) [16, 28].

### Abundance of bacteria in the metagenomes

In order to compare the abundance of bacteria in our datasets, we counted the number of reads recruited to bacterial concatenated contigs from the sequenced metagenomes. The number of reads was calculated using BLASTN, considering a minimum identity of 95% and a maximum e-value of 10^−3^ for filtering the results. The number of reads recruited per kb of the genome per Gb of the metagenomic dataset (RPKG) was used as a normalized value for comparing abundances.

## Results

Some summary statistics for each metagenome are shown in Additional file 1: Table S2. Assuming that a contig’s length is inversely proportional to bacterial diversity, the results of the assembly suggested that SMS is a not a very diverse niche. This observation was also confirmed by calculating alpha diversity indexes (Additional file 1: Table S2).

### Skin-mucus microbiome of wild European eel

GC profiles of the SMS-metagenomes from wild eels are shown in Additional file 1: Figure S4. The profiles vary according to waterbody salinity rather than to the eel specific habitat. Thus, wild eel SMS from river (water salinity below 1 g/L) was unimodal with a peak around 65% GC, that from estuarine waters of intermediate salinity (1–3 g/L) was bimodal, with an additional peak around 50% GC, and that from estuarine and wetland waters of higher salinity (7–10 g/L) was again unimodal but with a peak around 45% GC (Additional file 1: Figure S4).

**Fig. 1:**
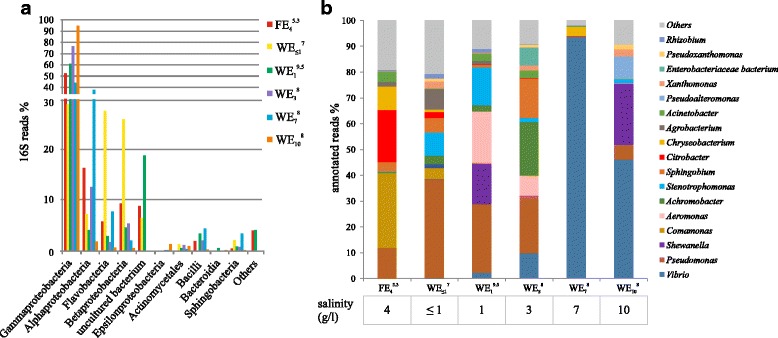
Main bacterial classes and genera detected in eel SMS-metagenomes. **a** Bacterial classes determined by 16S rRNA gene fragments classification in the metagenomics dataset. **b** Bacterial genera determined by taxonomic classification of all the reads in the metagenomes. Salinity of each habitat is shown at the bottom

We analyzed the bacterial taxa present in the SMS-metagenomes by using 16S reads (Fig. 1). We found that the SMS-microbiome was dominated by *Gammaproteobacteria*, whose proportion in the metagenomes ranged from 30% (wild eels from river) to 95% (wild eels from estuarine water of 7 g/L). *Flavobacteria* (1.6–30%), *Betaproteobacteria* (2.6–26%), and *Alphaproteobacteria* (5–24%) were other phyla dominant in the SMS-microbiome (Fig. 1a).

The main bacterial genera in the SMS-metagenomes are shown in Fig. 1b and Additional file 1: Figure S5. Results from gene annotation (Fig. 1b) and 16S reads (Additional file 1: Figure S5) were very similar. Remarkably, *Vibrio* was the dominant genus in the wild eel SMS-microbiome from estuary and wetland (46–93.5%) but decreased significantly in lake and river samples (9.8 and 0.2%, respectively). In these last samples, *Pseudomonas* (22–38%), *Stenotrophomonas* (10–14%), and *Achromobacter* (2–5%) were the most abundant genera (Fig. 1b).

**Fig. 2:**
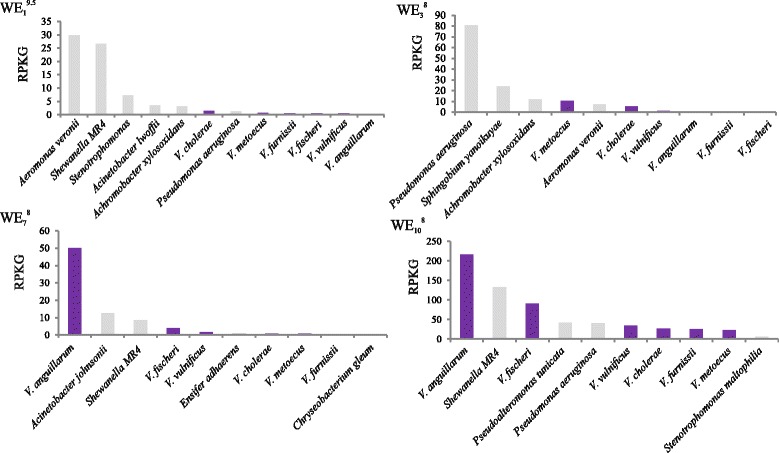
Main bacterial species detected in wild eel SMS metagenomes. The abundance of different species in the microbiomes is shown. The result was normalized dividing by the size of the genome (kb) and the dataset (Gb) (RPKG). *Aliivibrio* (*Vibrio*) *fischeri* was considered inside the *Vibrio*

Genome abundance based on metagenomic fragment recruitment showed differences in species composition related to the origin of the sampled eels (waterbody salinity) (Fig. 2). Thus, *Vibrio anguillarum*, *V. metoecus* (a recently described species closely related to *V. cholerae*) [29], and *V. cholerae* were the most abundant *Vibrio* species in mucus from waterbodies of 7–10 g/L, 3 g/L, and 1 g/L, respectively (Fig. 2). *Vibrio fischeri* (currently *Aliivibrio fischeri*), *Vibrio furnissii*, and *V. vulnificus* were also associated to eel mucus in the Nature Parks (Fig. 2). Abundance values for *V. vulnificus* were very low in all the metagenomes with the exception of WE_10_^8^ (Fig. 2). All the mentioned species, except the symbiotic species *Al. fischeri*, are well-known human and/or fish pathogens. Among them, *V. anguillarum* and *V. vulnificus* are pathogenic for a wide range of teleosts, including eels [30, 31]. In both species, the virulence in fish relies on the virulence plasmids pJM1 and pVvBt2, respectively [32, 33]. However, we did not find any evidence of the presence of any of these plasmids (or their genes) in our metagenomes.

**Fig. 3:**
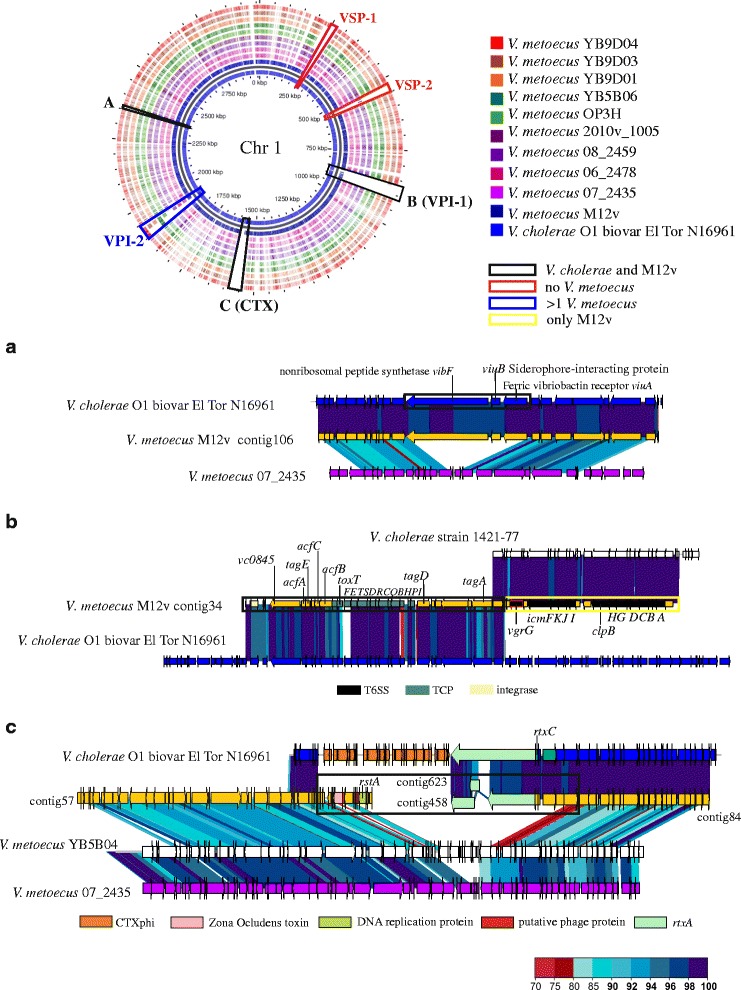
*V. metoecus* M12v BLAST atlas. Chromosome I of *V. cholerae* O1 biovar ElTor N16961 (reference) was compared with all available *V. metoecus* genomes and the strain M12v (sequenced in this study). Each ring represents a single color coded strain. Genomic islands are highlighted. Black boxes represent islands shared by *V. cholerae* and *V. metoecus* M12v while blue and red are used when ≥ 1 and 0 *V. metoecus* strains had the island, respectively. Islands found only in M12v (black box **a**, **b**, and **c**) were plotted using BLASTX against reference and the most similar *V. metoecus* strain

In parallel to the metagenomic study, we also isolated *V. metoecus* from SMS of wild eels captured in Ebro-Delta (salinity 3 g/L)*.* We selected the major yellow colony on TCBS agar, which was green on VVM agar (isolate M12v), and sequenced its genome. The average nucleotide identity (ANI) between M12v and *V. metoecus* RC341 was 98.24%, suggesting that isolate M12v belonged to the species *V. metoecus*. The comparison of M12v contigs to published genomes of *V. metoecus* (five strains isolated from a brackish coastal pond on the US east coast [34], as well as four clinical strains) and with that of *V. cholerae* O1 biovar El Tor N16961, highlighted that M12v shared with *V. cholerae* O1 but not with *V. metoecus* (i) two phage genes, *zot* (Zonula Occludens toxin), belonging to CTXphi and *rtsA* (encoding a phage protein), belonging to RS1 [35, 36]; (ii) most of the genes present in the pathogenicity island VPI-1, including the loci for the toxin co-regulated pilus (TCP) and the regulator ToxT; (iii) the loci for El Tor-RTX (Repeat in Toxin) biosynthesis, modification, and transport; and finally, (iv) most genes for vibriobactin biosynthesis and transport, including its outer membrane receptor ViuA (Fig. 3). Other genes present in M12v and *V. cholerae* El Tor but absent in strains of *V. metoecus* were genes for a nickel transport system, a phosphotransferase system, and a monovalent cation/proton antiporter (Additional file 1: Figure S6). Remarkably, downstream of the TCP locus we found a Type 6 secretion system (T6SS) absent in both *V. metoecus* strains and *V. cholerae* El Tor but present in *V. cholerae* strain 1421-77, a no-O1/no-O139 clinical isolate previously sequenced. Finally, M12v lacked the pathogenicity islands VSP-1 and -2 as the rest of *V. metoecus* strains and presented some genes of the VPI-2 in common with most of *V. metoecus* strains (Fig. 3; Additional file 1: Figure S7).

Other accidental pathogens identified in the SMS-microbiome from wild eels were *Pseudomonas aeruginosa*, *Stenotrophomonas maltophilia*, *Achromobacter xylosoxidans*, and *Aeromonas veronii*, the last being considered as potential eel pathogen [37].

### Mucus-attached vs free-living bacteria

**Fig. 4:**
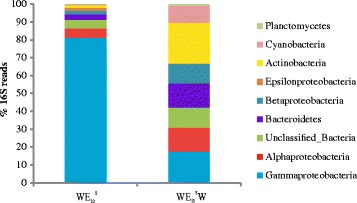
Main bacterial classes detected in eel SMS- and W-metagenomes. High level taxa determined by 16S rRNA gene fragment classification of the two metagenomics datasets. WE_10_^8^ (eel SMS); WE_10_^8^W (water)

The community composition of the attached mucus microbiome to that of the surrounding water (W) in the case of Ebro Delta ecosystem (water salinity 10 g/L) was quite different in spite of samples being taken at the same time (Fig. 4). Such differences have been previously shown by us in SMS-microbiome of eels from Lake Albufera as well [8]. While *Actinobacteria* (20%) and *Gammaproteobacteria* (17%) were the dominant taxa in the W-microbiome, however, the genus compositions of *Gammaproteobacteria* were quite different (Additional file 1: Figure S8). Only *Pseudomonas* was present in the same proportions in both metagenomes (around 2%) while *Vibrio* was remarkably overrepresented in SMS (32 vs 1% in water). The percentage of 16S reads that could not be assigned to any known genera was higher in W-metagenome (82%) than in SMS-metagenome (55%) (Additional file 1: Figure S8).

We analyzed by culture the waterbody where *V. anguillarum* was not detected by sequencing in spite of being the dominant species in eel SMS. The most abundant colony recovered on TCBS plates corresponded to *V. vulnificus* (identification by API20E plus PCR against *vvhA*). The isolated colonies were identified as belonging to biotypes 1 and 2 because they were yellow on VVM agar [38]. One of the isolates was sequenced to examine presence/absence of virulence genes. We detected an *rtxA1* gene that appears to be a new type emerged after hybridization between the two main *rtxA1* genes described in *V. vulnificus rtxA1*_*1*_, present in the most virulent biotype 1 strains and *rtxA1*_*3*_, and present in biotype 2 strains [39] (Additional file 1: Figure S9).

### Functional differences between mucus-attached and free-living microbiome

**Fig. 5:**
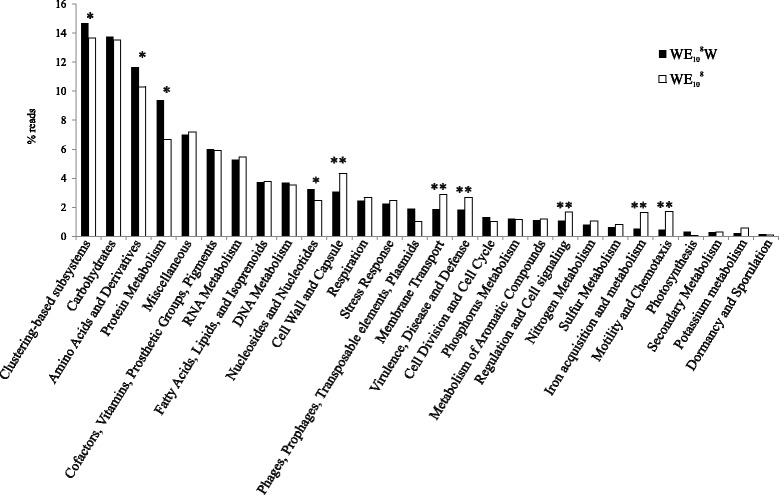
Differences in functional capacities detected in eel SMS- and W-metagenomes. The presence of each category is presented as percentage of reads assigned from the whole annotated metagenome. WE_10_^8^ (eel SMS); WE_10_^8^W (water).*Category visually over-represented in water; **Category visually over-represented in mucus

We analyzed and compared the functional capabilities of SMS- and W-metagenomes by classifying metagenomic reads in functional categories [40] (Additional file 2: Table S1). At the highest classification level, cell-wall/capsule, membrane-transport, virulence/disease/defense, regulation/cell-signaling, iron-acquisition/metabolism, N/S/K-metabolism, and motility/chemotaxis were the over-represented categories in SMS-metagenome (Fig. 5). On the other hand, amino-acids/derivatives, protein metabolism, nucleosides/nucleotides, phages/prophages/transposable-elements/plasmids, and photosynthesis were the over-represented ones in W-metagenome.

Specific searches in the classification for cholera toxin yielded no results in either metagenome. However, Ace (accessory cholera toxin) and ZOT (zonula occludens toxin), both present in CTXphi phage of *V. cholerae* [41] were found in the SMS-microbiome. Importantly, all types of bacterial secretion systems were overrepresented in mucus samples, e.g., T6SS which is known to be advantageous for bacterial competition [42, 43] (Additional file 1: Figures S10 and S11).

We analyzed in depth the SMS-metagenomes assembled contigs to look for other toxin genes. We found a paralog for *rtxA* gene in *Ali. fischeri*. We also found two genes for toxins in two *Pseudomonas* contigs: ExoU and ToxA which were 100 and 99% identical to their respective copies in *P. aeruginosa* WH-SGI-V-07317.

**Fig. 6:**
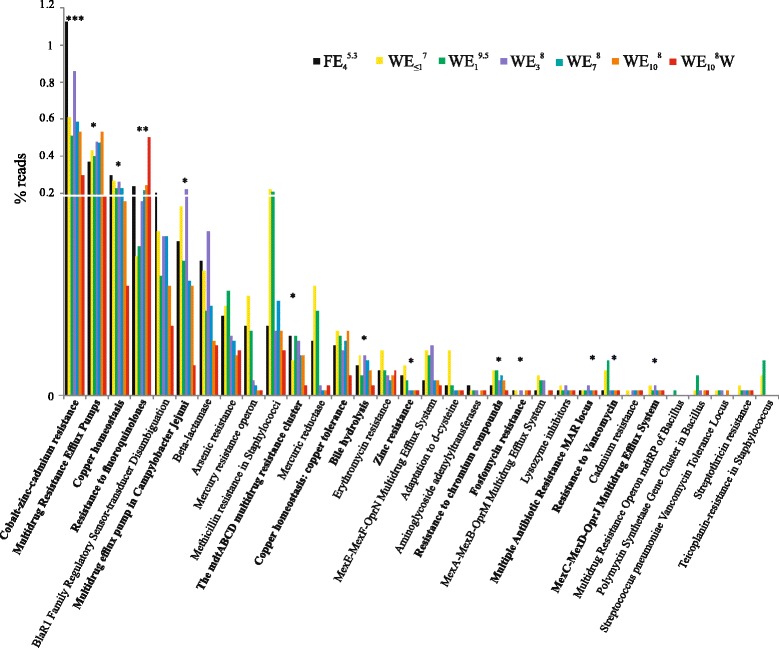
Abundance of gene categories involved in resistance to antibiotics and toxic compounds in wild eel SMS and W-metagenomes. An asterisk at the top indicates at least two times more abundance in SMS-metagenome than in W-metagenome; two asterisks indicate at least two times more abundance in W-metagenome than in SMS-metagenome; and three asterisks indicate at least two times more abundant in farmed eel SMS than in wild eel SMS

Finally, we looked for differences in antibiotic resistance genes between SMS- and W-metagenomes (Fig. 6). Genes for cobalt(Co)-zinc(Zn)-cadmium(Cd), multidrug efflux pumps, copper (Cu), aminoglycoside adenylyltransferases, chromium compounds, fosfomycin resistance, lysozyme inhibitors, MAR (Multiple Antibiotic Resistance) locus, and bile hydrolysis were duplicated in SMS-metagenome (Fig. 6). On the contrary, resistance to fluoroquinolones was clearly dominant in W-metagenome.

### SMS-microbiome from farmed vs wild eels

In order to find differences between the SMS-microbiomes of farmed vs wild eels, we used SMS-metagenome from wild (WE_3_^8^) and farmed eels (FE_4_^5.3^) as they had similar salinities (Table 1). At high taxonomic levels, *Alphaproteobacteria* was present in higher levels in mucus from farmed eels (Fig. 1a). However, at genus level, *Vibrio*, *Aeromonas*, and *Xanthomonas* were not present in farmed eel, microbiome while *Comamonas*, *Citrobacter*, and *Chryseobacterium* were significantly abundant in farmed eels (Fig. 1b; Additional file 1: Figure S5). Curiously, farmed eels maintained some of the genera found in wild eels from estuary and wetlands, such as *Pseudomonas*, *Acinetobacter*, *Stenotrophomonas*, and *Sphingobium* in significant proportions, (Additional file 1: Figure S5). This suggests these genera could be part of the eel SMS-resident microbiome.

Moreover, farmed eels microbiome presented less antimicrobial-related genes than wild metagenomes, with only one exception, resistance to Zn and Cd (Fig. 6). In fact, methicillin- and vancomycin-resistance clearly were overrepresented in SMS from wild eels.

### MGE in the attached microbiome: the flexible metagenome

**Fig. 7:**
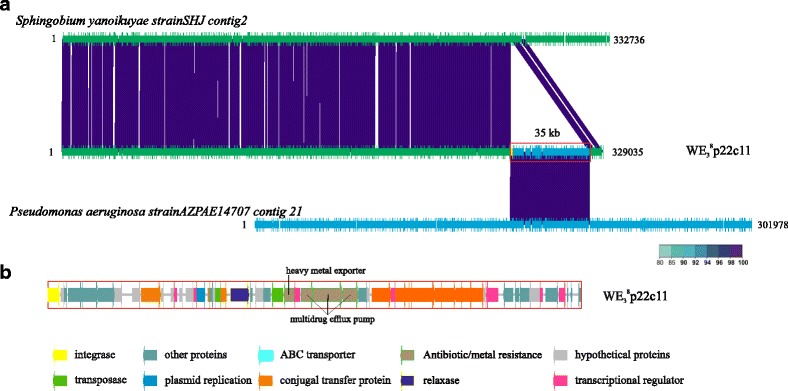
Horizontal gene transfer of an ICE between different genera. **a** The contig was compared against the genomes of the most similar strains in NCBI. **b** The pMGE (putative MGE) was re-annotated and classified as an ICE. Minimum identity of 80 was used to filter the BLASTN results

We looked for MGE in metagenomes WE_3_^8^ and WE_10_^8^ as well as in nine previously published metagenomes (Additional file 1: Table S1) by using the workflow presented in Additional file 1: Figure S3. PCA analysis from HUP values showed contigs assigned to the same bacterial species but with and without pMGE as clearly distinguishable clusters (Additional file 1: Figures S12-S14). Regarding GC variation, the interchange of DNA material between GC-rich populations was easily detected since GC content of the genera varied from 50 to 70%. For example, *Pseudomonas* and *Sphingobium* apparently exchanged long fragments of DNA (> 35 kb) (Fig. 7). However, we could not find inserted sequences looking at the GC content in the samples mainly composed by *Vibrio*.

We found 118 and 12 cpMGE in WE_3_^8^ and WE_10_^8^, respectively, and none in the rest of SMS-metagenomes probably because very few contigs larger than 10 kb were assembled. The protocol was useful to highlight the presence of lytic phages and prophages previously described in these metagenomes (Additional file 1: Figures S12-S14) [12]. Once we identified the cpMGE in the metagenome, we re-annotated the genes inside using HHpred and, then, classified them according to gene content. Additional file 1: Table S3 shows the confirmed contigs and the type of pMGE that contained in each case. Only 34 of the pMGE could be clearly classified: integrative and conjugative elements (ICE), integrons, CRISPR, prophages (described in 43), and transposons. These MGE were detected in the contigs assigned to the dominant genus *Vibrio*, *Pseudomonas*, *Sphingobium*, *Achromobacter*, *Enterobacterium*, and *Aeromonas* (Additional file 1: Table S3 and Figure S15). Most of them encoded genes for colonization and resistance to drugs and heavy metals.

We also identified various MGE in *Pseudomonas* contigs, which had been previously related to pathogenicity islands encoding two toxins associated with resistance to innate immune system (phagocytosis), the toxins ToxA and ExoU [44] (Additional file 1: Figure S16). The genetic context of both genes in our metagenomes was also compatible with a pathogenicity island.

Finally, we found evidences of exchange of long DNA fragments among bacteria. For example, we found sequences from different genera inserted within *Sphingobium* and *Achromobacter* contigs. We focused our attention on one contig, which contained a putative ICE (WE_3_^8^C14) of significant size (47 kb) with a 100% identity and coverage to a contig of *Ps. aeruginosa strain AZPAE14707* and *AZPAE14724* (Additional file 1: Figure S17). These strains were isolated from the respiratory tract of a patient in Greece and from an intra-abdominal tract infection in Italy, respectively. Moreover, this ICE was highly similar to other *Pseudomonas* sequences from different ecosystems (Additional file 1: Figure S17). Three genes encoding a multidrug efflux pump were inserted within this ICE. Other interesting contigs were WE_3_^8^C164 and WE_3_^8^C158 that were part of the same ICE in *Achromobacter* (Additional file 1: Figure S18). Surprisingly, resistance to three different components (mercuric, arsenic, and multidrug efflux pump) were encoded in this MGE. The putative ICE (> 48 kb) separated in these contigs also hit *Pseudomonas* genomes in the databases including one that also matched the previously mentioned one (WE_3_^8^C11). *Pseudomonas*, *Sphingobium*, and *Achromobacter* have similar GC content, and their abundance in our metagenomes was also similar.

## Discussion

The hypothesis underlying this work is that the fish SMS could constitute an appropriate environment for evolution and emergence of new mucosal pathogens of aquatic origin. These mucosal surfaces are exposed to water and are similar in composition, structure, and defense mechanisms to the human intestinal mucosa [45]. As a first approach to test this hypothesis, we have analyzed the microbiome of a euryhaline fish, the European eel, whose surface is covered by a thick layer of mucus [46]. This species inhabits distinct environments, from the open sea to rivers and lakes, and even can be grown in farms.

The eel natural SMS-microbiome was dominated by *Gammaproteobacteria* while the W-microbiome did not present any clear dominant class being rich in unknown bacteria and, according to [12] also by phages. Further, the natural SMS-microbiome varied in bacterial composition, both qualitative and quantitatively, according to the salinity of the waterbody where eels were captured. Thus, the genus *Vibrio* was dominant in natural SMS-microbiome from estuarine eels at salinities between 7 and 10 g/L while a mixture of genera predominated in estuarine, lake, and river eels at salinities ≤ 3 g/L. We identified *V. anguillarum*, *V. vulnificus*, *V. fischeri*, *V. furnissii*, *V. metoecus*, and *V. cholerae* as the dominant vibrios in all the eel SMS samples, which suggests that they are an important part of the resident natural microbiome. In general terms, each one of these species of resident vibrios increased its proportion in SMS-microbiome at the salinity values closest to its optimal value for growth [47–49]. Thus, the dominant *Vibrio* species in natural SMS-microbiome was *V. anguillarum* at salinities between 7 and 10 g/L, *V. metoecus* at salinity of 3 g/L, and *V. cholerae* at salinity of 1 g/L. Interestingly, while *V. anguillarum* was dominant in the SMS-microbiome, it was not detected in water, suggesting that bacterial species composition in fish SMS is not a simple reflection of that of surrounding water. Instead, it appears that SMS attracts and selectively concentrates specific members of the aquatic microbiome, among which there are multiple vibrios. We hypothesized that such “specialized” bacteria, although could be present in low concentration in the water, because of these “specialized capabilities” would attach to and multiply in the fish SMS, a part of them being finally established as resident microbiota. Supporting this hypothesis, we found that alpha diversity indices decreased four times in the attached microbiome in comparison to the water microbiome.

In parallel, we analyzed by culture the waterbody negative for *V. anguillarum* contigs in spite of this bacterium being the dominant one in eel SMS-microbiome. The recovered bacteria belonged to *V. vulnificus*. The genome of the selected isolate lacked the plasmid pVvBt2, essential to cause fish vibriosis [38, 50, 51] and was positive for cellobiose fermentation (biotype 3 strains are negative), and therefore, it corresponded to a biotype 1 strain. The most striking feature of this genome was that it contained a new type of *rtxA1*. *V. vulnificus* produces seven types of RTX toxins, all of them classified within the subfamily MARTX (multifunctional autoprocessing repeats-in-toxin) [52]. MARTX present two common external modules, containing the repeated sequences, together with a specific internal module, and containing a unique combination of effector domains responsible for the toxic activity of the protein (nine specific domains have been described) [52]. The new type of MARTX_Vv_ (MARTX type VIII) seems to be a hybrid from types I (produced by the most human virulent biotype 1 strains) and type III (biotype 2 strains), both involved in resistance to phagocytosis by immune cells and, at least type III, in resistance to amoeba, one of the main bacterial predators in the environment [53]. It has been proposed that *rtxA1*_*3*_ emerged by recombination between a new variant of *rtxA1*, arriving with the plasmid pVvBt2, and the resident chromosomal gene [54]. We proposed that *rtxA1*_*8*_ could have emerged in the environment by recombination between *rtxA1*_*1*_ and *rtxA1*_*3*_ following an HGT event. In any case, this finding strongly supports that new variants of these modular toxins are continuously emerging in the environment, in this case in water.

*V. vulnificus* biotype 2 is highly virulent for eels and has caused the closure of many eel farms due to massive mortality [55]. To control eel vibriosis, some farmers decided to use freshwater instead of brackish water in farm facilities, but this measure resulted in the emergence of new serovars [56]. Subsequently, most of the intensive eel farms closed and the remaining ones combined low salinity with low pH to eradicate vibriosis. This seems to be quite effective since no *Vibrio*, including *V. vulnificus*, was detected in the SMS-microbiome from farmed eels (artificial SMS-microbiome). In fact, the artificial SMS-microbiome was dominated by bacterial species resistant to acid pH such as members of *Comamonas*, *Citrobacter*, and *Chryseobacterium* and, in particular, by *Comamonas testosteroni*, a bacterium resistant to low pH and antibiotics [57, 58]. This result is quite interesting, because it confirms that the outcome of infectious diseases in aquatic animals in captivity can be modified by a change in a selected water physicochemical parameter(s) (such as pH) that results in a major change in the composition of fish SMS-microbiome.

We also analyzed the differences in heavy metals and drug resistance genes between the artificial and natural SMS-microbiome. Resistance to Co, Zn, and Cd were significantly overrepresented in the artificial SMS-metagenome. Fish farmers frequently use antibiotics and metal containing products to prevent fouling, to feed, and to treat fish in order to limit the spread of infections [59]. The overrepresentation of these resistance genes in mucus from farmed eels stresses the importance of substituting these practices by methods such as immunostimulants in diet or vaccination to prevent not only the disease but also the spread of resistance genes to the environment.

Among the rest of *Vibrio* pathogenic species found in the wild eel SMS-microbiome, *V. metoecus* is perhaps the least studied. Originally, *V. metoecus* was described as a non-virulent clone isolated from Chesapeake Bay (USA) [60]. This species is the closest to *V. cholerae*. In fact, both species share the aquatic habitat and have been co-isolated from Oyster Ponds in the USA [34]. In this work, we found both species together with other vibrios and when eels were fished in water of 0.3 g/L salinity; *V. metoecus* was the dominant *Vibrio* species. This finding suggests that fish SMS constitutes a new environment from which *V. metoecus* could be co-isolated with *V. cholerae*, the first species dominating at 0.3 g/L water salinity.

*V. metoecus* was successfully isolated from SMS-samples, and one strain was sequenced, M12v. The M12v genome showed strong evidences of HGT from *V. cholerae* O1 ElTor since it contained (i) the VPI-1, an island that encodes the ability to colonize human intestine [61], (ii) some CTXphi and RS1 phage genes [35, 36], (iii) an RTX toxin cluster [62], and (iv) a locus for vibriobactin biosynthesis and transport [63, 64]. In all these traits, our eel mucus isolate was different from the rest of *V. metoecus* strains, all of them from water and extra-intestinal infections. The presence of all these *V. cholerae* genes, especially those involved in intestinal colonization (siderophore and TCP), strongly suggests that M12v would be better adapted to mucus than the rest of *V. metoecus* strains sequenced so far. In consequence, *V. metoecus* avirulent clones could become virulent by the oral route after acquiring *V. cholerae* virulence genes when both species co-exist in the same environment and are under selective pressure.

To find out what forces were acting to select the best adapted bacteria or to favor HGT events lending expansion of the best mucus-adapted recombinants, we compared the functionalities of SMS- and W-metagenomes. The genes that were particularly enriched in the SMS allow us to predict the following functional categories as essential for successful mucus colonizers: (i) Biofilm (exopolysaccharide production, sigma-dependent biofilm formation, VieSAB signal transduction system, etc.), (ii) bacterial communication (quorum sensing, autoinducer-2-transport/processing etc.), (iii) bacterial competition (bacteriocin-like peptides, ABC transporter peptide and type T6SS, etc.), (iv) adherence (colonization-factor-antigen-1, curli, accessory-colonization-factors, *Campylobacter*-adhesion etc.), (v) resistance to humoral innate immunity (including nutritional immunity) (lysozyme resistance, bile hydrolysis, multidrug efflux pumps, siderophore biosynthesis and transport, hemin-uptake, etc.), (vi) resistance to phagocytosis: (RTX toxins, two lytic toxins of *Pseudomonas* and for types II, III, and IV secretion systems), (vii) resistance to lysis by phages: (CRISPR systems), and (viii) resistance to heavy metals and drugs: Co-Zn-Cd, Cu, multidrug efflux pumps, antibiotic resistance. Remarkably, most of the genes found in each category could be classified within the SMS-virulome and SMS-resistome, respectively.

Regarding the SMS-resistome, this would contain the genes encoding resistance to the heavy metals Co, Zn, Cd, and Cu, which were clearly over-represented in SMS-metagenomes in comparison to water. Although the sampled habitats are protected from human pollution, there are numerous rice fields in the surrounding areas [65, 66], and in consequence, this contamination would have probably derived from agricultural activities. Remarkably, some of these resistances were detected to be encoded in pMGE, mostly ICEs, in the analyzed metagenomes. It has been reported that the mucin of the mucus covering the fish epidermis can bind heavy metals by electrostatic forces and concentrate them on the fish surface [67], which could favor the exchange of these pMGEs by HGT events. Finally, these genes were overrepresented in artificial vs natural SMS-metagenome, confirming the relationship between its frequency in a sample and its degree of contamination.

Curiously, we also found drug-resistance genes that were overrepresented in W-metagenome from Nature Park and that corresponded to resistance to fluoroquinolones. Fluoroquinolones can be excreted by humans and animals into hospital or municipal sewage [68], resist the wastewater treatment plants, and remain for decades in the environment [69–71]. Two large hospitals are located in the surroundings of the Ebro Delta, which was declared protected area in August 1983, what could explain the prevalence of fluoroquinolone resistance genes in the W-metagenome from this location.

With regard to SMS-virulome, it appears to contain all the genes found in our metagenomes for tissue colonization, from genes for bacterial adhesins to genes for resistance to mucosal innate immunity such as those involved in iron uptake. Among them, there were genes encoding cytolytic toxins that could be putatively involved in resistance to phagocytosis as it is MARTX_Vv_ type III [53]: (i) an *rtxA* gene found in *Ali. fischeri* contigs; (ii) *ace* (accessory cholera enterotoxin) and *zot* genes found in *V. cholerae* contigs, originally described as part of the phage CTXphi; and finally, (iii) *exoU* and *toxA* genes found in *Pseudomonas* contigs, practically identical to their paralogs in *P. aeruginosa* WH-SGI-V-07317. All these toxins are secreted by different secretion systems, i.e., ToxA and ExoU are injected by T3SS [72]. In accordance, we found that all types of secretion systems were overrepresented in mucus samples, including T6SS, which is known to be essential for bacterial competition in the environment [42, 43]. Interestingly, some of these genes were found to be associated to pMGE suggesting that HGT events occur in mucus under the selective pressure of innate immunity and, probably, natural predators.

When the genes presumptively acquired by our SMS *V. metoecus* isolate from *V. cholerae* were compared with the functional categories over-represented in mucus, we found that the cluster of genes for the T6SS, the TCP pilus biogenesis, the vibriobactin biosynthesis and uptake, and the RTX toxin and its transport could be classified in the categories, iii (Bacterial competition), iv (Adherence), v (Resistance to mucosal innate immunity), and vi (Resistance to phagocytosis), respectively. All these findings suggest that fish SMS selects phenotypic traits that favor fitness in mucus and that these traits could be advantageous for these bacteria if they accidentally arrive to the human intestine.

## Conclusions

In conclusion, we have obtained multiple evidences from metagenomic, genomic, and culture in favor of the hypothesis on the role of fish SMS as an important niche for mucosal pathogen evolution in nature. First, SMS concentrates bacteria present in water, in particular vibrios; second, selects those bacteria with particular abilities to attach, resist innate immunity, and compete with other bacteria; and third, favors the exchange of genes encoding these functions. Particularly interesting is that we have isolated a new variant of *Vibrio* with intermediate properties between *V. metoecus* and *V. cholera*e O1 El Tor, abilities that are encoded in pathogenicity islands and phages. It appears that genetic exchange takes place primarily in the fish SMS and that this niche, conceivably, provides the selective pressures for acquisition and maintenance of the colonization and competition associated virulence phenotypes, phenotypes that could collaterally mediate bacterial fitness in the human intestine.

## Additional files

Additional file 1: Table S1. Metagenomes used to detect MGE. **Table S2.** General data for each metagenome and alpha diversity. **Table S3.** Contigs with MGE detected using the methodology described in Fig. 1. **Figure S1.** Sampling points, location and description. **Figure S2.** From nature to the laboratory: skin mucus sampling from wild eels and DNA extraction. **Figure S3.** Mobile genetic elements (MGE) detection workflow diagram. **Figure S4.** %GC content profiles of the eel’s SMS- and W-metagenomes. **Figure S5.** Wild eel’s versus farmed eel’s SMS metagenomes. **Figure S6.** *V. metoecus* M12v BLAST atlas. **Figure S7.** Schematic representation of VPI-2 in M12v. **Figure S8.** Main bacterial genera detected in eel’s SMS- and W-metagenomes. **Figure S9.** *rtxA1* gene comparison. **Figure S10.** Differences in functional capacities between SMS-associated and water microbiomes. **Figure S11.** Differences in membrane transport functional categories between SMS-associated and water microbiomes. **Figure S12.** PCA analysis of hexanucleotide usage pattern (HUP) of water metagenomes. **Figure S13.** PCA analysis of hexanucleotide usage pattern (HUP) of metagenomes associated to different hosts. **Figure S14.** Hexanucleotide usage pattern (HUP) distribution of the attached microbiome to epidermal mucus of European eels in WE_3_^8^. **Figure S15.** Contigs with pMGE. Genes of interest are colored differently. **Figure S16.** MGE in a contig of *Pseudomonas*. **Figure S17.** Distribution of an ICE identified in contigWE_3_^8^C14 between *Pseudomonas* strains. **Figure S18.** Exchange of long DNA stretches between genera with similar %GC of the genome. (DOCX 4303 kb)

Additional file 2: Table S1. Abundance and comparison of functions in water and skin-mucus surface microbiomes. (XLSX 646 kb)

## Abbreviations

cpMGE: Contigs identified as putative mobile genetic elements
HGT: Horizontal gene transfer
HUP: Hexanucleotide usage pattern
ICE: Integrative and conjugative element
MARTX: Multifunctional autoprocessing repeats-in-toxin
MGE: Mobile genetic elements
PBS: Phosphate buffer saline
PCA: Principal component analysis
RPKG: Reads recruited per kb of the genome per Gb of the metagenomic dataset
sgMGE: Signature genes for mobile genetic elements
SMS: Skin-mucosal-surface
T6SS: Type 6 secretion system
TCBS: Thiosulfate-citrate-bile salts-sucrose
TCP: Toxin co-regulated pilus
VVM: *Vibrio vulnificus* medium
W: Water
