# Supplementary material for: Wild eel microbiome reveals that skin mucus of fish could be a natural niche for aquatic mucosal pathogen evolution
Source: Microbiome. 2017 Dec 21;5:162. doi: 10.1186/s40168-017-0376-1 (PMC5740887; doi:10.1186/s40168-017-0376-1)
Supplement: Supplementary file 1 — Metagenomes used to detect MGE. Table S2. General data for each metagenome and alpha diversity. Table S3. Contigs with MGE detected using the methodology described in Fig. 1. Figure S1. Sampling points, location and description. Figure S2. From nature to the laboratory: skin mucus sampling from wild eels and DNA extraction. Figure S3. Mobile genetic elements (MGE) detection workflow diagram. Figure S4. %GC content profiles of the eel’s SMS- and W-metagenomes. Figure S5. Wild eel’s versus farmed eel’s SMS metagenomes. Figure S6. V. metoecus M12v BLAST atlas. Figure S7. Schematic representation of VPI-2 in M12v. Figure S8. Main bacterial genera detected in eel’s SMS- and W-metagenomes. Figure S9. rtxA1 gene comparison. Figure S10. Differences in functional capacities between SMS-associated and water microbiomes. Figure S11. Differences in membrane transport functional categories between SMS-associated and water microbiomes. Figure S12. PCA analysis of hexanucleotide usage pattern (HUP) of water metagenomes. Figure S13. PCA analysis of hexanucleotide usage pattern (HUP) of metagenomes associated to different hosts. Figure S14. Hexanucleotide usage pattern (HUP) distribution of the attached microbiome to epidermal mucus of European eels in WE3 8. Figure S15. Contigs with pMGE. Genes of interest are colored differently. Figure S16. MGE in a contig of Pseudomonas. Figure S17. Distribution of an ICE identified in contigWE3 8C14 between Pseudomonas strains. Figure S18. Exchange of long DNA stretches between genera with similar %GC of the genome. (DOCX 4303 kb) [file 40168_2017_376_MOESM1_ESM.docx]

**Supplementary Tables**

Table S1. Metagenomes used to detect MGE.

| Name | Sample | Sequencing technology | Nº contigs >10 kb | Access number |
| --- | --- | --- | --- | --- |
| WE_3_^8^ | Host-associated (mucus) | Illumina | 1321 | SRA185006, SRA185006 |
| WE_10_^8^ | Host-associated (mucus) | Illumina | 391 | SRR1586370 |
| DCM | Aquatic (Mediterranean) | Illumina | 720 | SRR037008 |
| HumanDiabetes | Host-associated (gut) | Illumina | 63 | SRR341581 |
| Mouse | Host-associated (feces) | Illumina | 210 | 4535626.3, 4535627.3 |
| SeaUrchin | Host-associated (gut) | Illumina | 330 | ERR895166 |
| Moose | Host-associated (gut) | Illumina | 587 | ERS624611 |
| Indian | Aquatic (Lake) | Illumina | 211 | ERS433966 |
| Swedish | Aquatic (Lake) | Illumina | 138 | ERS433967 |

Table S2. General data for each metagenome and alpha diversity.

|  | **FE^4^_5.3_ | **WE_≤1_^7^ | **WE_1_^9.5^ | WE_7_^8^ | WE_3_^8^ | WE_10_^8^ | WE_10_^8^W |
| --- | --- | --- | --- | --- | --- | --- | --- |
| Dataset size (Mb) | 70 | 49.8 | 45.3 | 1300 | 3939 | 3622 | 959 |
| Number of sequences | 101780 | 91574 | 70510 | 12325876 | 25287346 | 46064452 | 12490702 |
| Average length (bp) | 634,6 | 486,1 | 587,5 | 87.4 | 88.25 | 78.6 | 76.9 |
| Number of contigs >1Kb (Mb) | 6074 (8.2) | 8217 (16.6) | 1609 (20.8) | 1016 (23.08) | 5050 (45.13) | 12069 (35.8) | 2608 (6) |
| Average contig length (bp) | 1354 | 2027.7 | 1291.5 | 2272 | 8938 | 2970.2 | 2302 |
| Number of contigs >10Kb (Mb) | 0 | 0 | 0 | 7 (0.3) | 699 (33.26) | 415 (11.2) | 27 (0.56) |
| Average contig length (bp) | 0 | 0 | 0 | 43310.6 | 47590.1 | 27052 | 20993 |
| Viral contigs (Mb) | 218 (0.30) | 67 (0.12) | 79 (0.11) | 1 (0.22) | 33 (0.43) | 181 (0.86) | 127 (0.6) |
| Average contig length (bp) | 1399.6 | 1890 | 1413.8 | 220068 | 13066.2 | 4786.9 | 4770.9 |
| Viral contigs > 10Kb (Mb) | 0 | 0 | 0 | 1 | 12 (0.36) | 21 (0.49) | 14 (0.39) |
| Average contig length (bp) | 0 | 0 | 0 | 220068 | 29921.8 | 23795.6 | 27867.9 |
| alpha diversity | 132.73 | 401.08* | 101.46 | 157.54 | 50.99 | 107.72 | 422.59 |

* Rarefaction curve did not reach a plateau

**Sequenced using 454. The rest of metagenomes were sequenced using Illumina.

Table S3. Contigs with MGE detected using the methodology described in Figure 1.

| Genus | Contig  length  (kb) | MGE classification | MGE length (kb) | | Virulence/  Resistance/  Competence/  genes | Detected  by |
| --- | --- | --- | --- | --- | --- | --- |
| *Pseudomonas* | WE_3_^8^C1 (893.5) | Integron (*Cupriavidus*) | ND | Chemotaxis,  acriflavin resistance | | GC |
|  |  | Integron | ND | Penicillin | | sgMGE |
|  |  | Integron | ND | Bleomycin  resistamce | | sgMGE |
|  |  | ND | ND | Virulence associated protein | | GC |
|  |  | Integron (*Pseudomonas*) | ND | * | | sgMGE |
|  | WE_3_^8^C2 (555.3) | Integron | 6 | Penicillin resistance | | sgMGE |
|  |  | Integron (*Pseudomonas*) | 47 | Toxin-antitoxin  system | | sgMGE |
| *Sphingobium* | WE_3_^8^C4 (520) | Prophage | 22.5 | Serine protease | | sgMGE |
|  | WE_3_^8^C11 (329) | ICE | ≈29.4 | Arsenic resistance | | sgMGE |
|  | WE_3_^8^C9 (360.7) | ND | 6.4 | Cobalt/zinc/cadmium transporter | | sgMGE |
|  | WE_3_^8^C93 (82) | Integron | ≥46 | Arsenic resistance | | sgMGE |
|  | WE_3_^8^C19 (208.8) | ICE | ≥70 | Siderophore  biosynthesis | | sgMGE |
|  | WE_3_^8^C22 (173.4) | ND | ≥23.5 | * | | sgMGE |
|  | WE_3_^8^C25 (151.2) | Integron | ≥55.8 | Penicillin resistance | | sgMGE |
|  | WE_3_^8^C29 (137.9) | Integron | 9.5 | Chitinase | | sgMGE |
|  | WE_3_^8^C30 (134.4) | Integron | ≥17.2 | Kanamycin resistance | | sgMGE |
|  | WE_3_^8^C38 (109.3) | ICE | 68.4 | * | | sgMGE |
|  | WE_3_^8^C49 (79.6) | Integron | 32.9 | Toxin-antitoxin  system | | sgMGE |
| *Achromobacter* | WE_3_^8^C24 (160.3) | ICE | 75.2 | Protease | | sgMGE |
|  | WE_3_^8^C37 (112.4) | ICE | 24 | Multidrug resistance pump | | sgMGE |
|  | WE_3_^8^C40 (112.4) | Transposon | 24.8 | Arsenic & mercuric resistance | | sgMGE |
|  | WE_3_^8^C158 (32.2) | Integron | 34.6 | Toxin-antitoxin  system | | sgMGE |
|  | WE_3_^8^C164 (31.8) | ICE | 32.4 | * | | sgMGE |
|  | WE_3_^8^C98 (78.9) | ND | 28 | Toxin-antitoxin  system | | sgMGE |
|  | WE_3_^8^C111 (41.9) | Prophage | 34.5 | * | | sgMGE |
|  | WE_3_^8^C190 (41.9) | ND | 2.8** | * | | annotation |
| *Aeromonas* | WE_3_^8^C55 (129.8) | Transposon | 14 | | Fimbrial operon | sgMGE |
| *Vibrio* | WE_10_^8^C8 (111.2) | ND | 9.2 | | * | annotation |
|  | WE_10_^8^C14 (94.5) | ND | 18 | | Fimbrial operon | annotation |
|  | WE_10_^8^C32 (56) | CRISPR | 0.2 | | * | sgMGE |
|  | WE_10_^8^C35 (52.8) | ND | ND | | * | sgMGE |
|  | WE_10_^8^C18 (76.9) | ND | ND | | * | sgMGE |
|  | WE_10_^8^C144 (23.7) | ND | ND | | * | sgMGE |
|  | WE_10_^8^C192 (18.6) | ICE | 125.9 | | Arsenic resistance | sgMGE |
|  | WE_10_^8^C278 (13.9) | ICE | ≥16 | | * | sgMGE |
| - | WE_3_^8^C57 (125.9) | Prophage | ND | | * | sgMGE |
| - | WE_10_^8^C168 (20.4) | ND | ≥6 | | Arsenic resistance | sgMGE |
| - | WE_10_^8^C96 (31) | ND | - | | RHS toxin | sgMGE |
| - | WE_10_^8^C386 (10.6) | Prophage | 43.1 | | Hemolysin | sgMGE |

ND, no determined

GC =detected by GC

sgMGE= detected by BLASTP using signature genes

**Supplementary Figures**


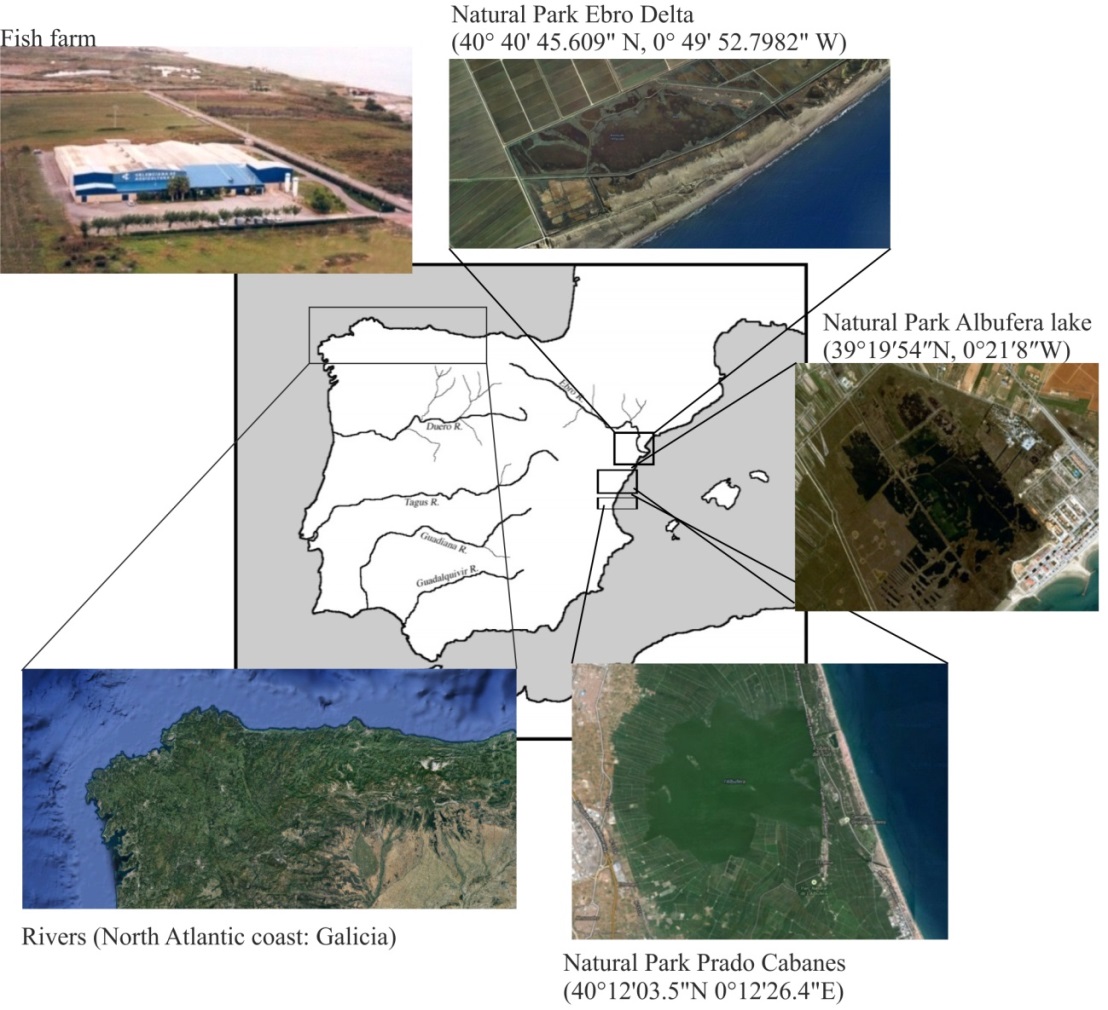


Figure S1. Sampling points, location and description. Albufera Nature Park is located on the Gulf of Valencia coast (Valencian Community, Spain) and contains a shallow (1 m depth on average) coastal lagoon that is the largest fresh-water lagoon in Spain. Because of the increased human activities in its densely populated surroundings, Albufera lagoon collapsed as a macrophyte-dominated lagoon and turned into a highly hypertrophic ecosystem with very dense phytoplankton populations primarily dominated by *Cyanobacteria* [1]. Ebro Delta Nature Park, one of the biggest Mediterranean wetlands, comprises the mouth of the Ebro river and surrounding areas (320 km^2^) and is located in south of Catalonia (Spain). Prat de Cabanes-Torreblanca Nature Park is a wetland located in the coastline of the municipalities of Cabanes and Torreblanca (Castellón, in the Valencian Community). We also sampled about 10,000 wild-eels (0.33 g each exemplar) captured from various rivers of the Atlantic coast of Spain (Galicia).


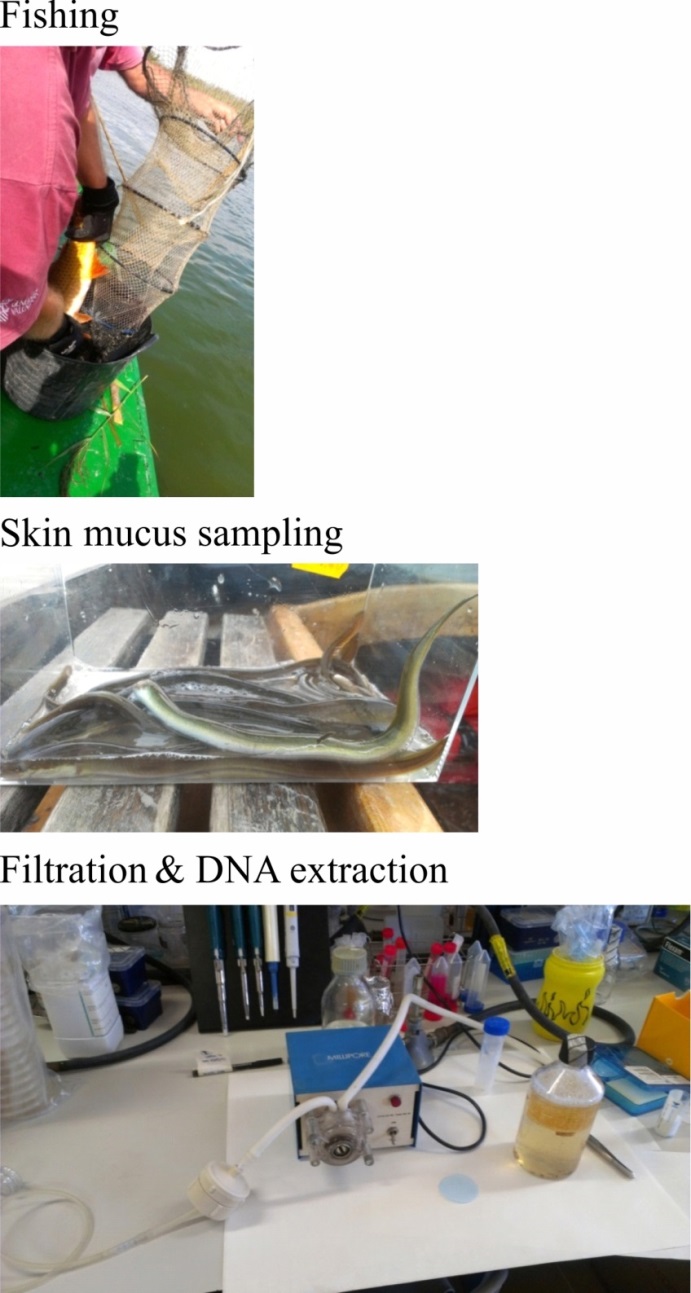


Figure S2. From nature to the laboratory: skin mucus sampling from wild-eels and DNA extraction. Nets were recovered 24 h after installation (fishing) and eels were deposited in fishbowls with PBS 1x sterile during 20 min (skin mucus sampling). The resulting solution was stored at 4ºC and transported to the lab where it was filtered to concentrate the microbial population. Then, DNA was extracted and sequenced (Filtration & DNA extraction). For details see Material and Methods section.


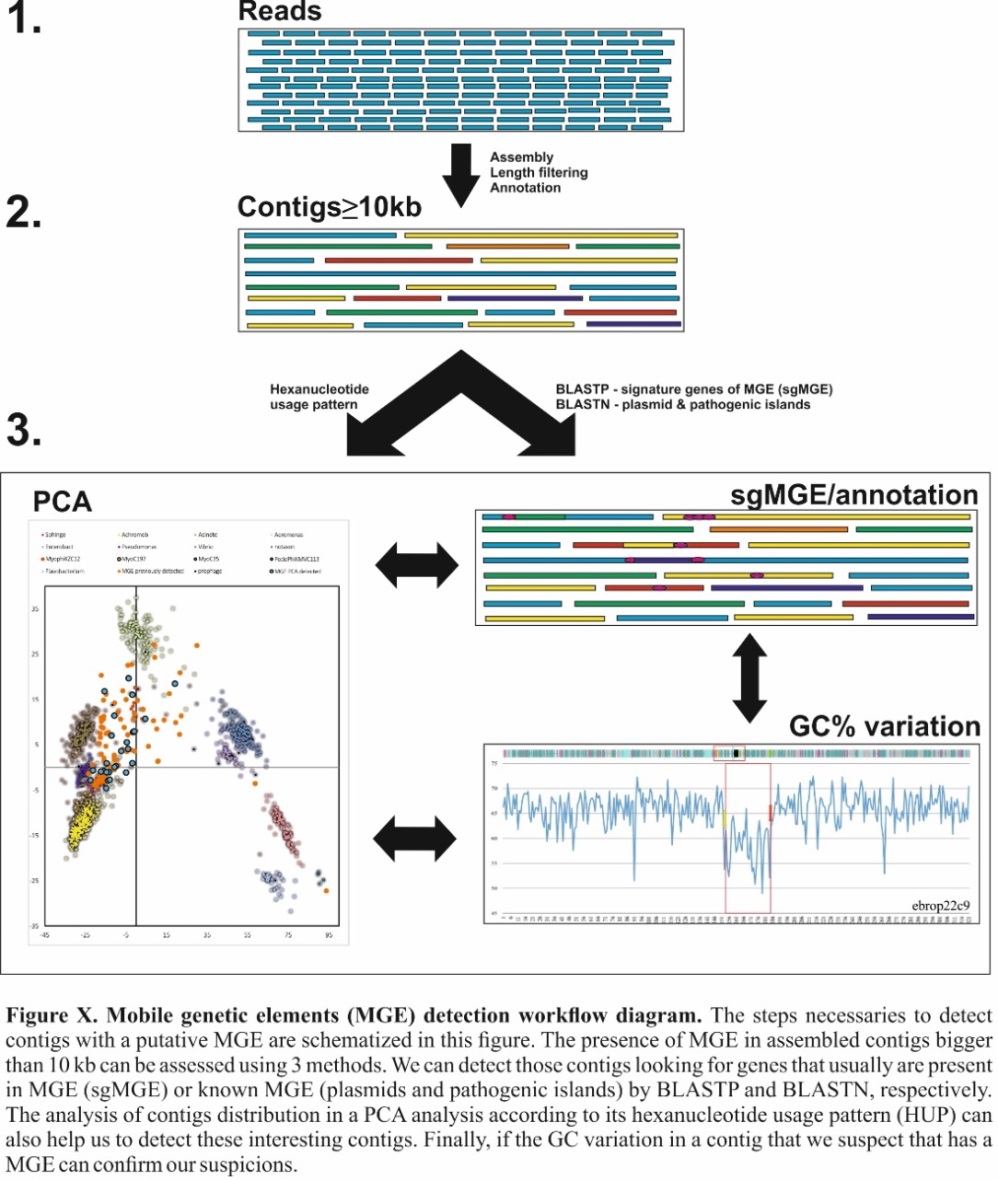


Figure S3. Mobile genetic elements (MGE) detection workflow diagram. The steps to detect contigs with a putative MGE are schematized in this figure. First we selected contigs bigger than 10 kb and looked for MGE-signature genes (sgMGE) as well as for known MGE (plasmids and pathogenic islands) by BLASTP and BLASTN, respectively. In parallel, a PCA analysis according to hexanucleotide usage pattern (HUP) was performed as well as a study of intra-contig GC variation.


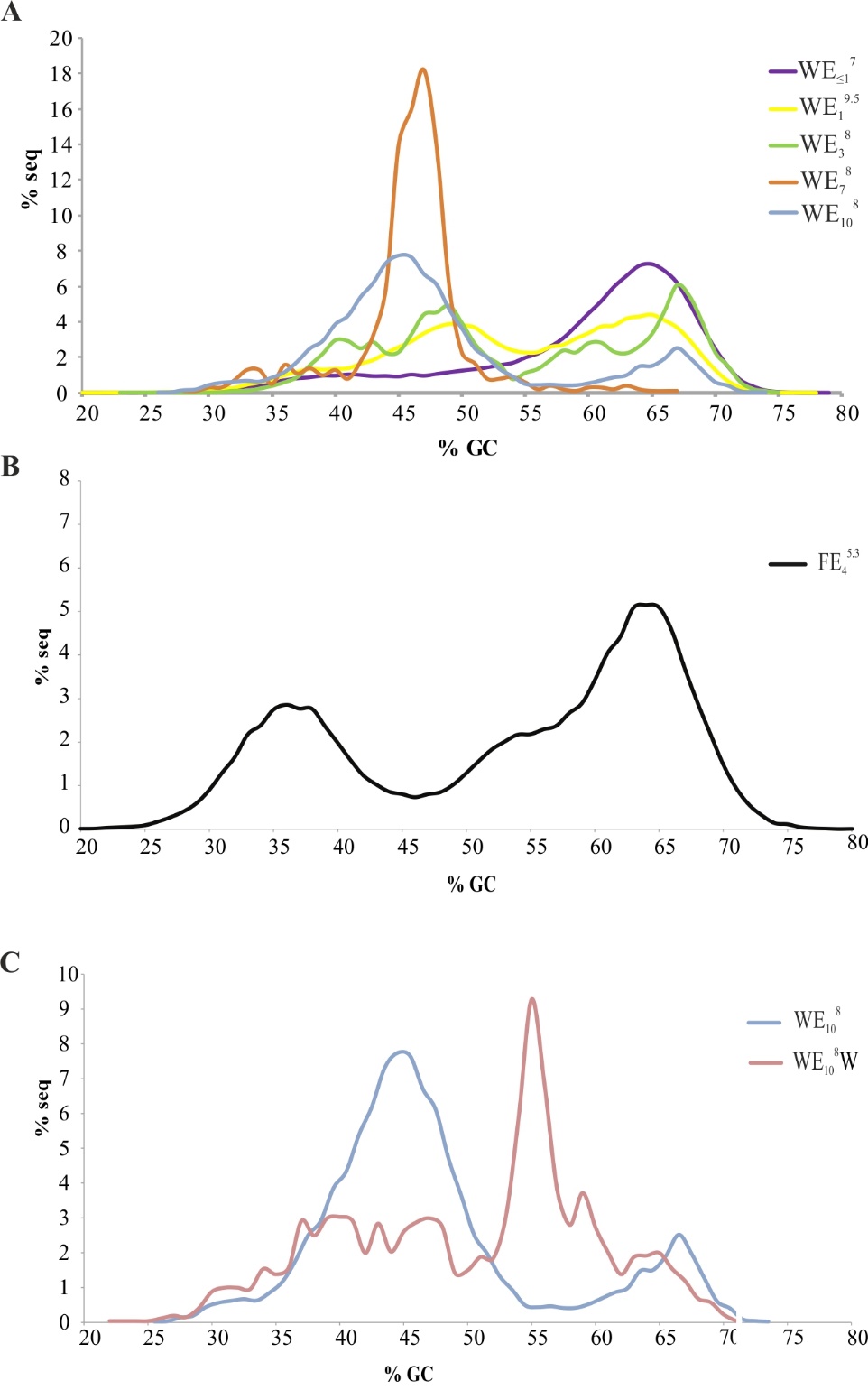
Figure S4. %GC content profiles of the eel’s SMS- and W-metagenomes. A, SMS-metagenomes from wild-eels; B, SMS-metagenomes from farmed-eels; C, SMS- (WE_10_^8^) and W- (WE_10_^8^W) metagenomes from Ebro Delta.


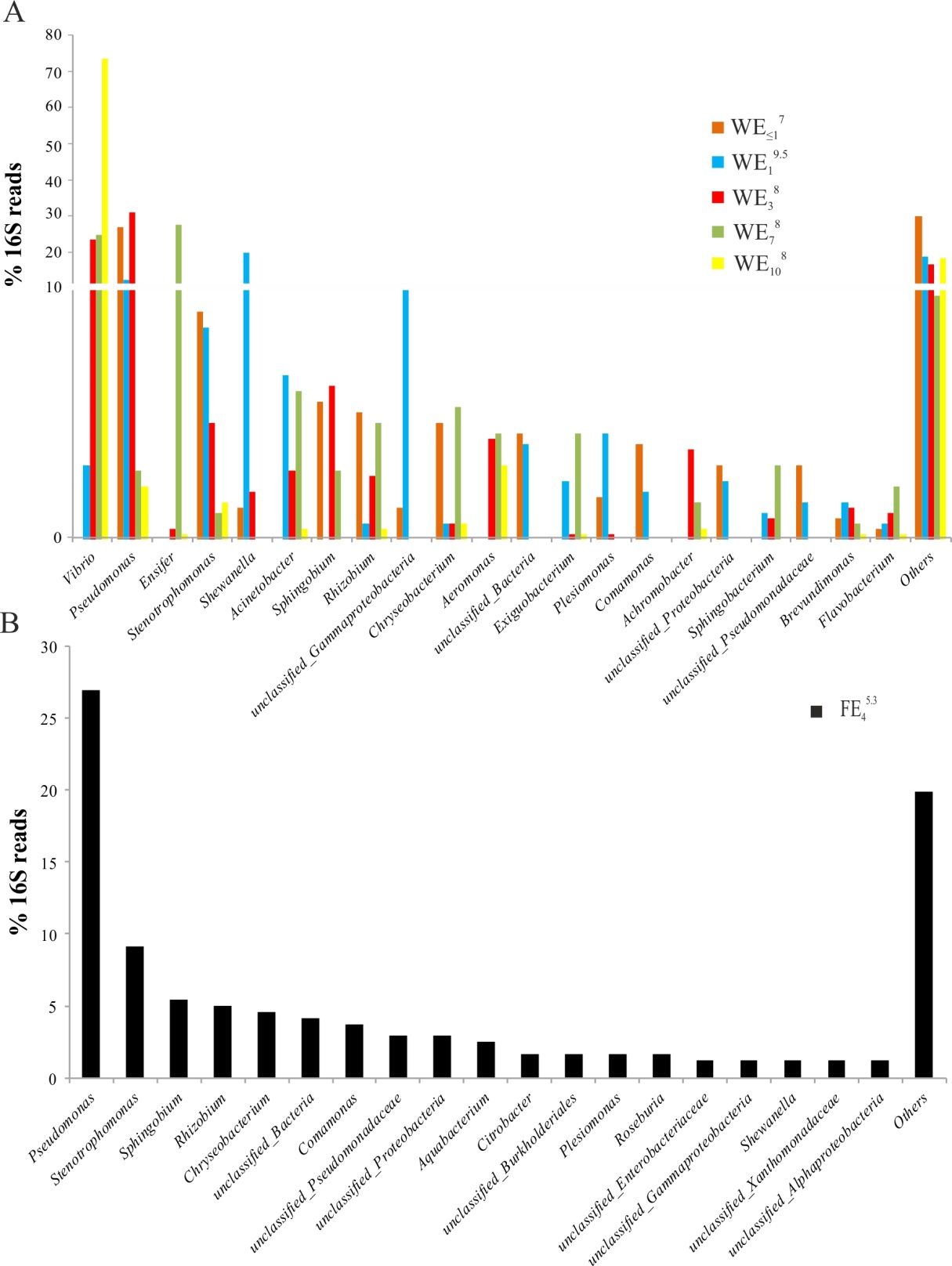


Figure S5. Wild-eel’s *versus* farmed-eel’s SMS metagenomes. The main bacterial genera in wild-eel’s SMS-metagenomes (A) and farmed-eel’s metagenomes were determined by 16S rRNA classification of the metagenomics dataset.


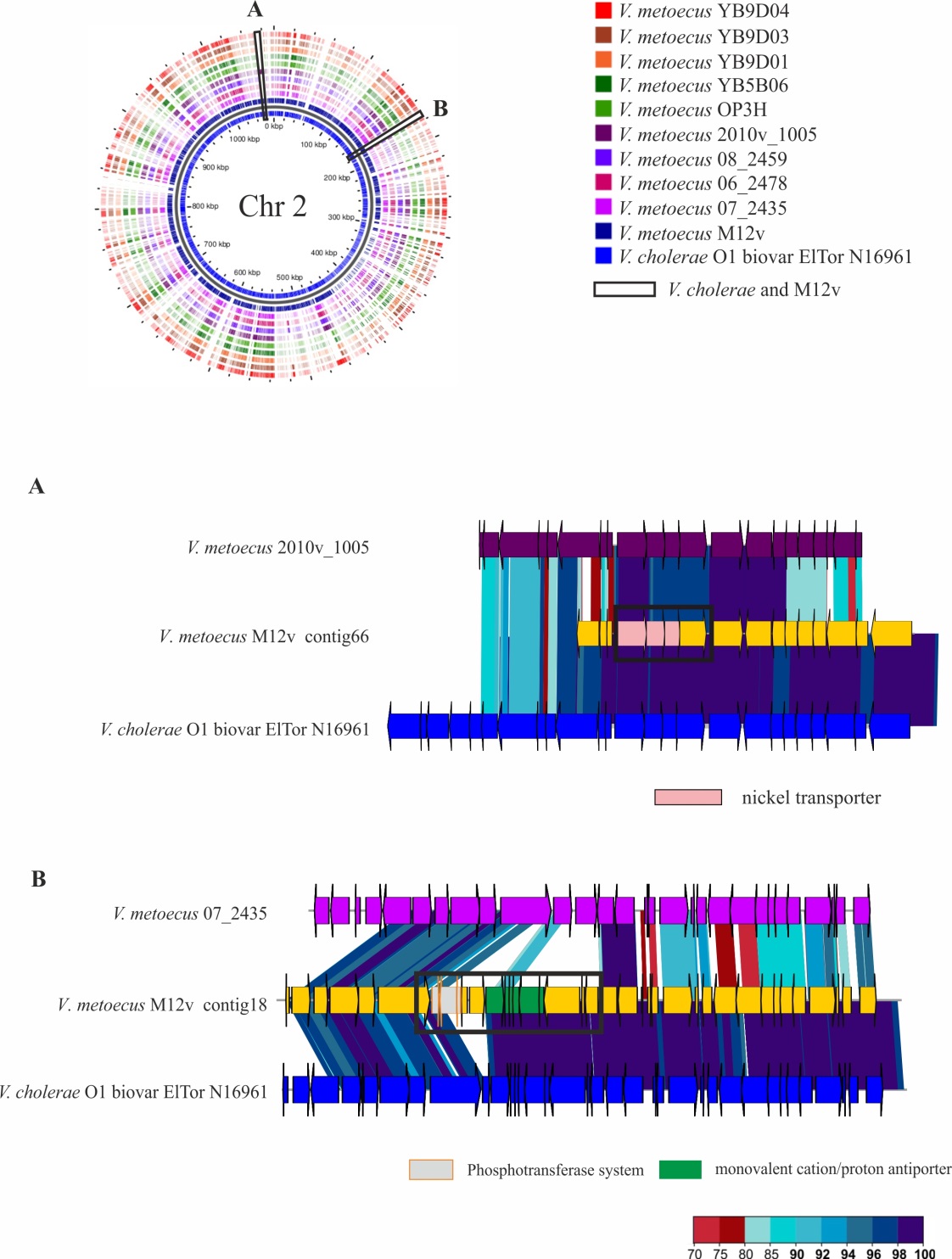


Figure S6. *V. metoecus* M12v BLAST atlas. Chromosome II of *V. cholerae* O1 biovar ElTor N16961 (reference) was compared with all available *V. metoecus* genomes and the strain M12v (sequenced in this study). Each ring represents a single color coded strain. Genomic islands are highlighted. Black boxes (A and B) represent islands shared by *V. cholerae* and *V. metoecus* M12v.


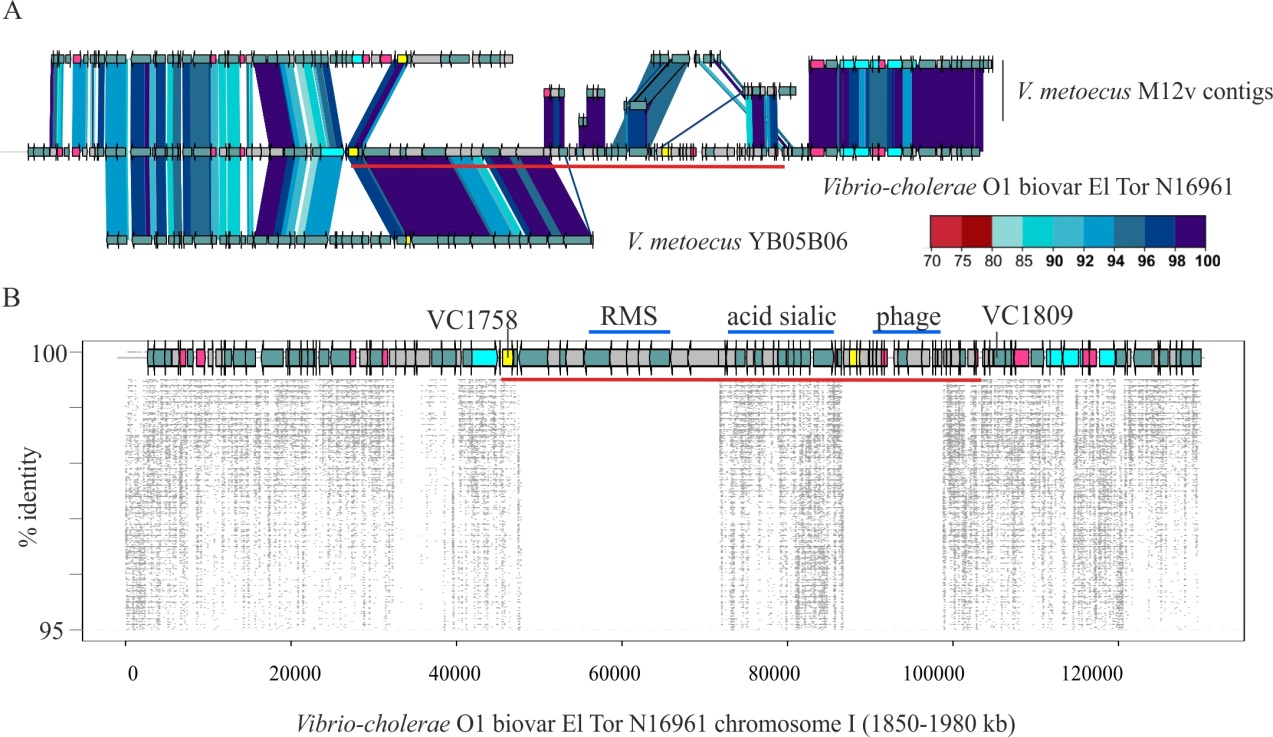


**Figure S7. Schematic representation of VPI-2 in M12v.** Contigs from M12v genome were compared to the pathogenic island VPI-2 in *V. cholerae* O1 biovar El Tor N16961 and *V. metoecus* using tBLASTX in A. In B, the reads from sequencing M12v genome were recruited against VPI-2. The region corresponding to VPI-2 and its three known regions are marked with a red or blue line, respectively. RMS, Restriction modification system; acid sialic, metabolism of sialic acid; phage, phage-like region.


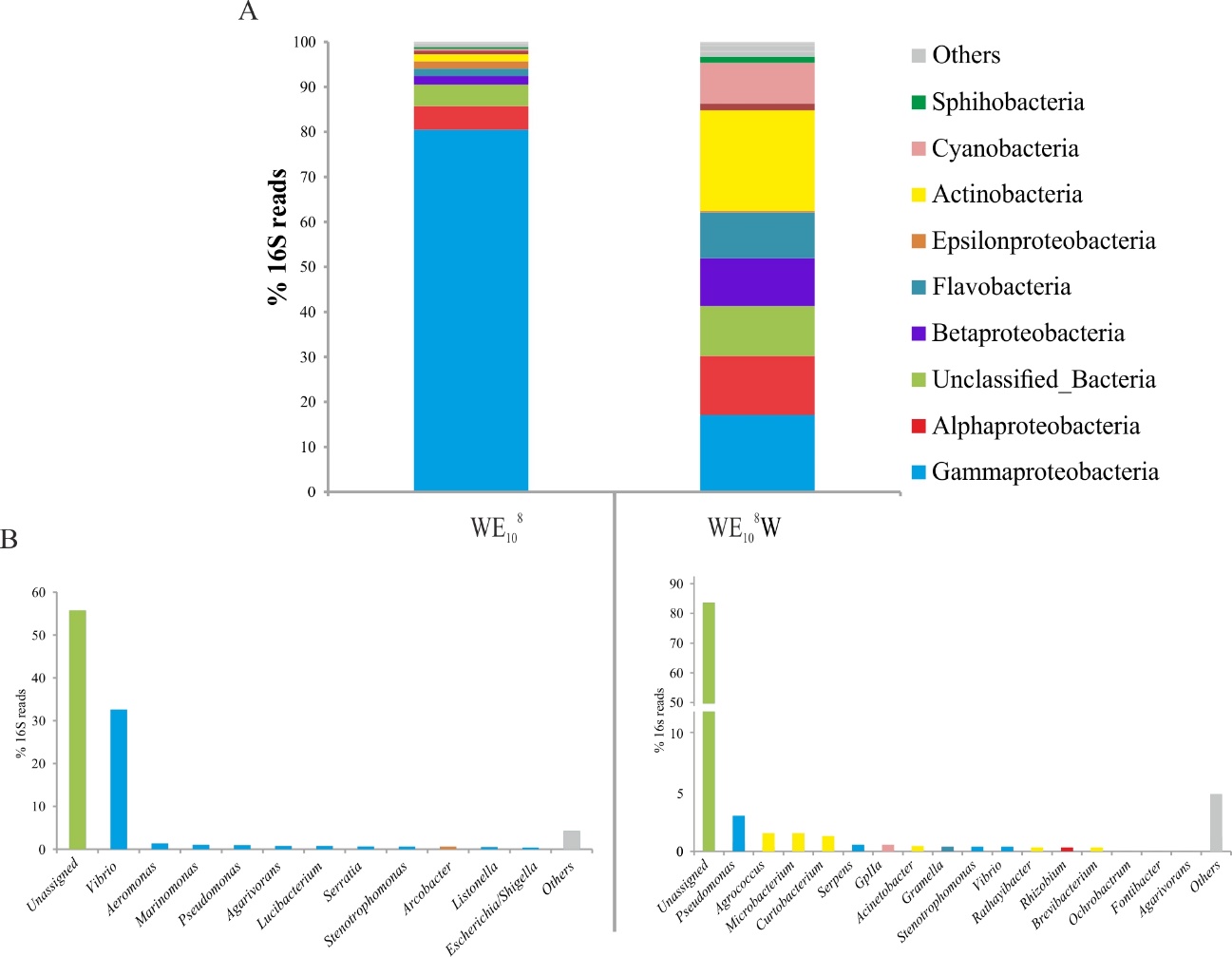


WE_10_^8^W

WE_10_^8^

**Figure S8.** **Main bacterial genera detected in eel’s SMS- and W-metagenomes**. Genus taxa determined by 16S rRNA gene fragment classification of the two metagenomics datasets. WE_10_^8^ (eel’s SMS); WE_10_^8^W (water). Color coded according to Class taxa.


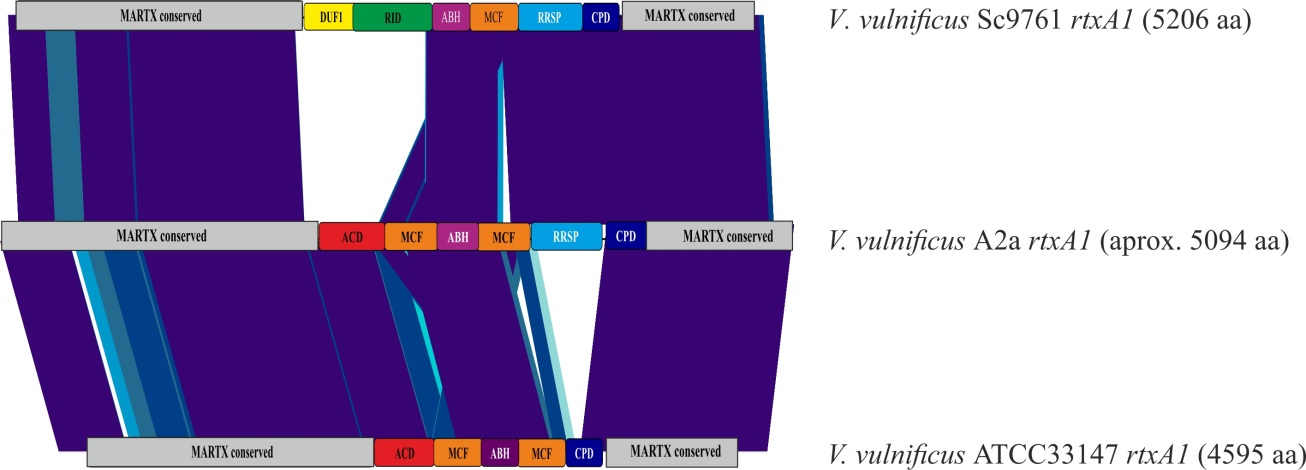


Figure S9. *rtxA1* gene comparison. The gene *rtxA1* sequenced in the *V. vulnificus* A2a, isolated from water (WE_10_^8^W) was compared to the most similar one in Genbank using tBLASTX. Minimum identity and length alignment of 80 and 150 were used, respectively. White narrows represent the three contigs in which the *rtxA1* was assembled. Strain Sc9761 is a biotype 1 strain that presents *rtxA1_1_* and strain ATCC33147 is a biotype 2 strain that presents *rtxA1_3_*. ACD: actin-cross-linking domain; MCF: Makes Caterpillars Floppy-like; RRSP: Ras/Rap1-specific protease; ABH: alpha-beta hydrolase; CPD: cysteine protease domain.


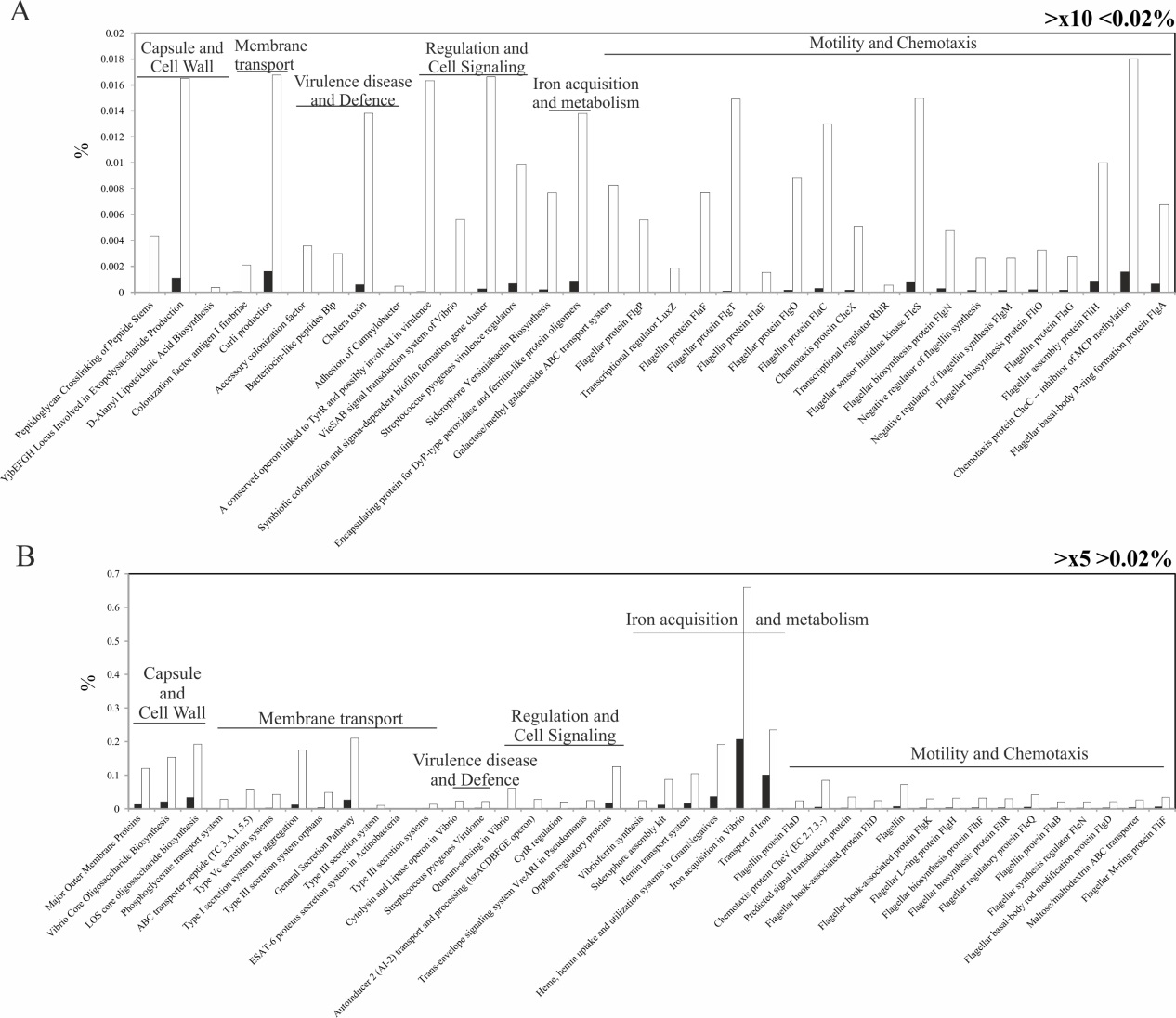


**Figure S10.** **Differences in functional capacities between SMS-associated and water microbiomes.** The genes overrepresented in the mucus according to MG-RAST pipeline are shown. These are included in the corresponding category using SEED classification. A, genes of presence <0.02 % but overrepresented at least 10 times. B, genes of presence >0.02% at least 5 times overrepresented. Black, water; White, mucus-associated.


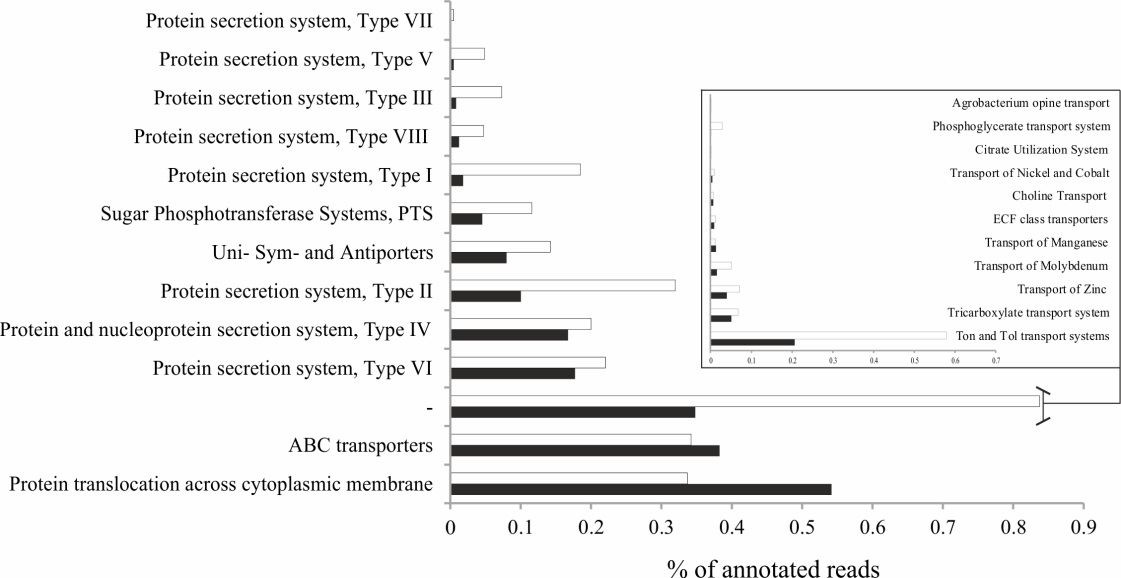


**Figure S11. Differences in membrane transport functional categories between SMS-associated and water microbiomes.** The abundance of functions categorized in “Membrane transport” was compared between Black, water; White, mucus-associated. The functions inside “-“ category were plotted in the upper box.


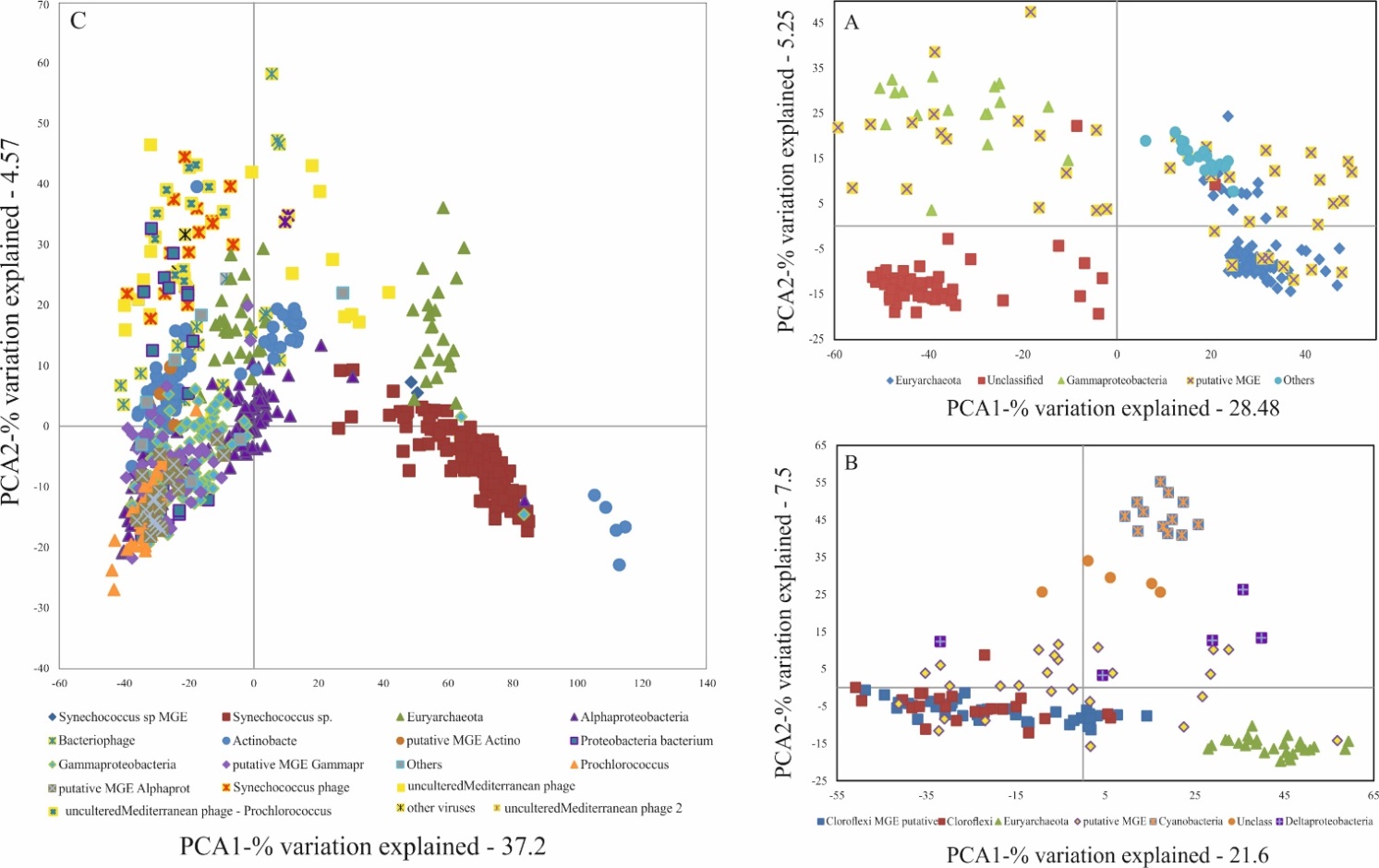


Figure S12. PCA analysis of hexanucleotide usage pattern (HUP) of water metagenomes. Contigs annotated to the most abundant genus and those that were considered putative MGEs were coloured differently. A, Indian lake; B, Swedish lake; C, Mediterranean Sea (DCM).


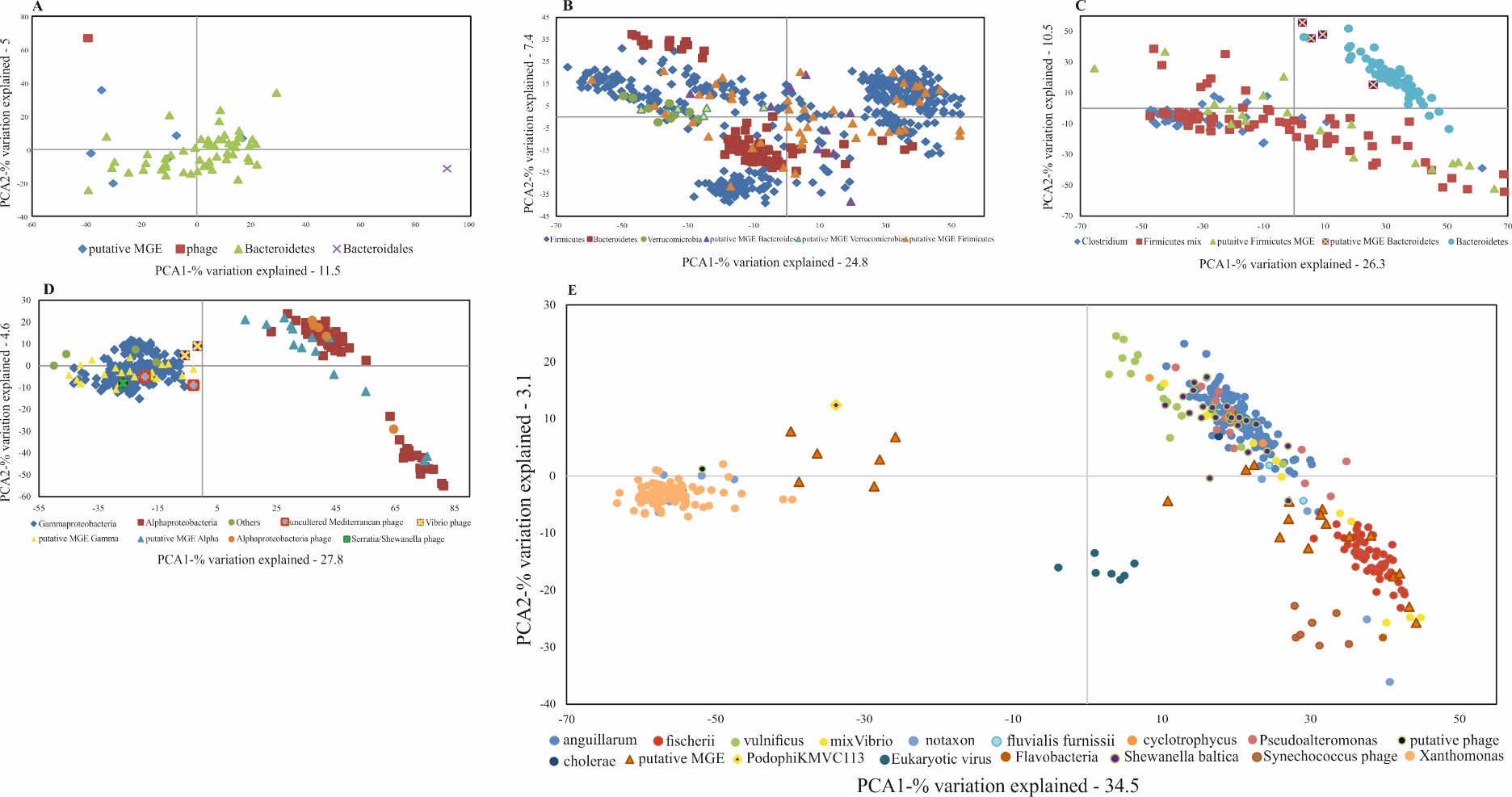


Figure S13. PCA analysis of hexanucleotide usage pattern (HUP) of metagenomes associated to different hosts. Contigs annotated to the most abundant genus and those that were considered putative mobile genetic elements were coloured differently. A, Human diabetes type 2; B, Moose; C, Mouse feces; D, Sea urchin; E, WE_10_^8^


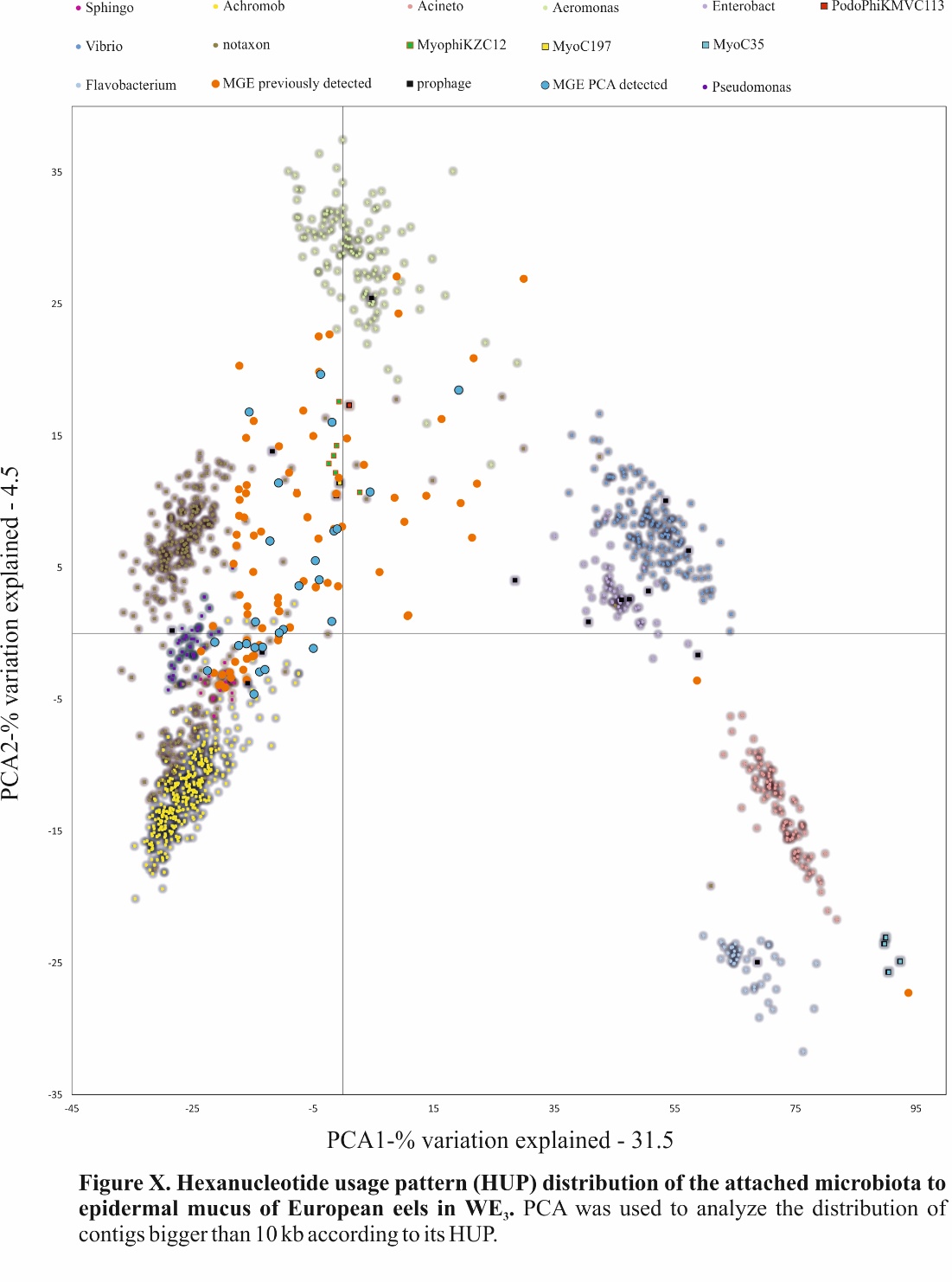


Figure S14. Hexanucleotide usage pattern (HUP) distribution of the attached microbiome to epidermal mucus of European eels in WE_3_^8^. PCA was used to analyze the distribution of contigs bigger than 10 kb according to its HUP.

**
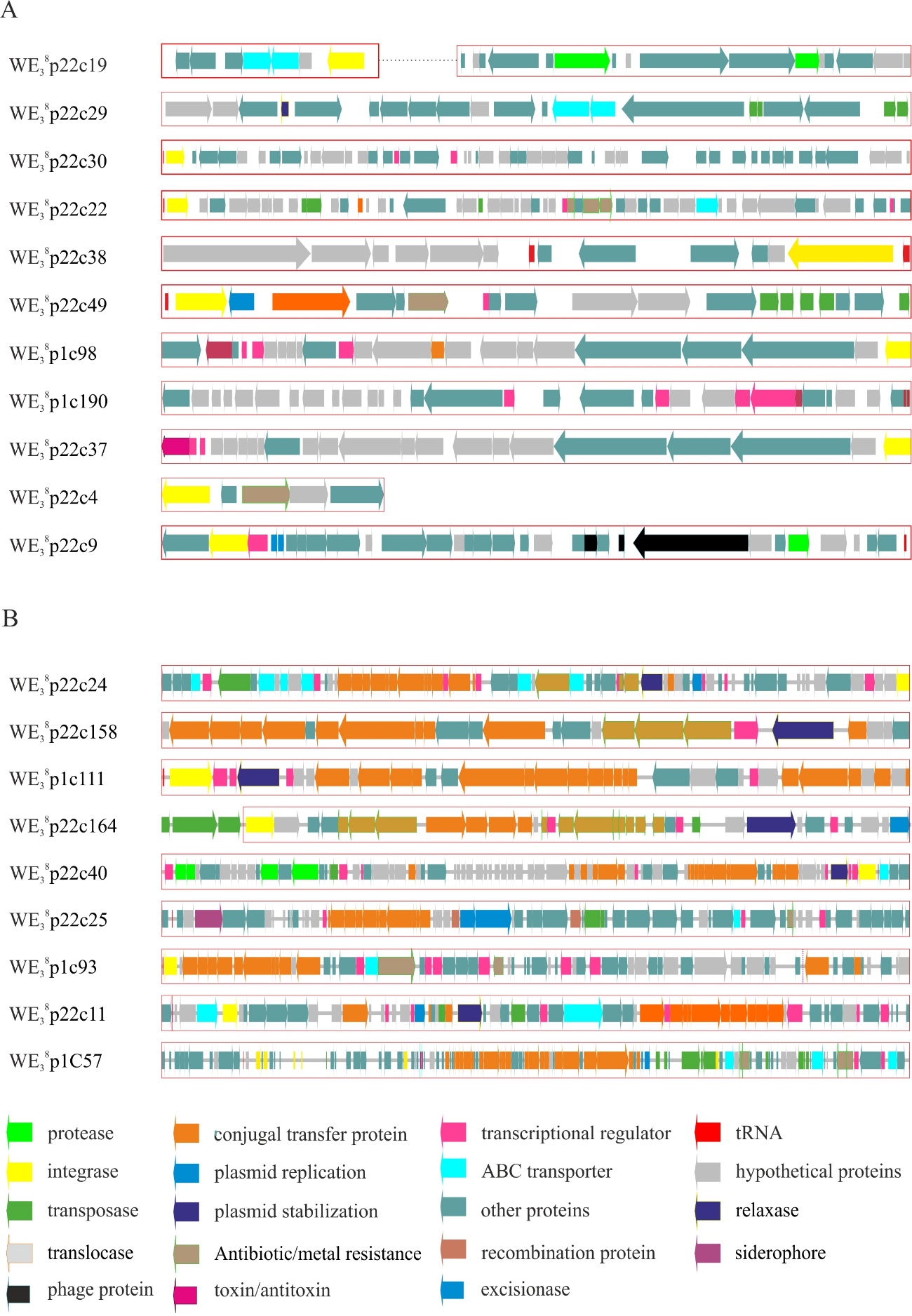
**

**Figure S15. Contigs with pMGE. Genes of interest are coloured differently.** A, pMGE that could not be ascribed to any type. B, pMGE that were classified as integrative and conjugative elements (ICE).

.


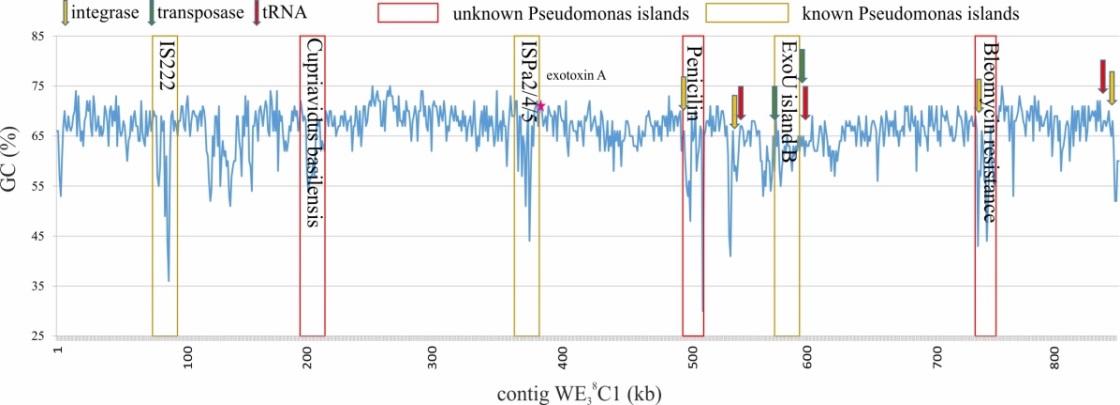


Figure S16. MGE in a contig of *Pseudomonas*. The GC percentage of each ORF is represented in the graph for the entire contig. The integrases, transposases and tRNA are indicated using arrows and detected islands are highlighted using colored boxes.


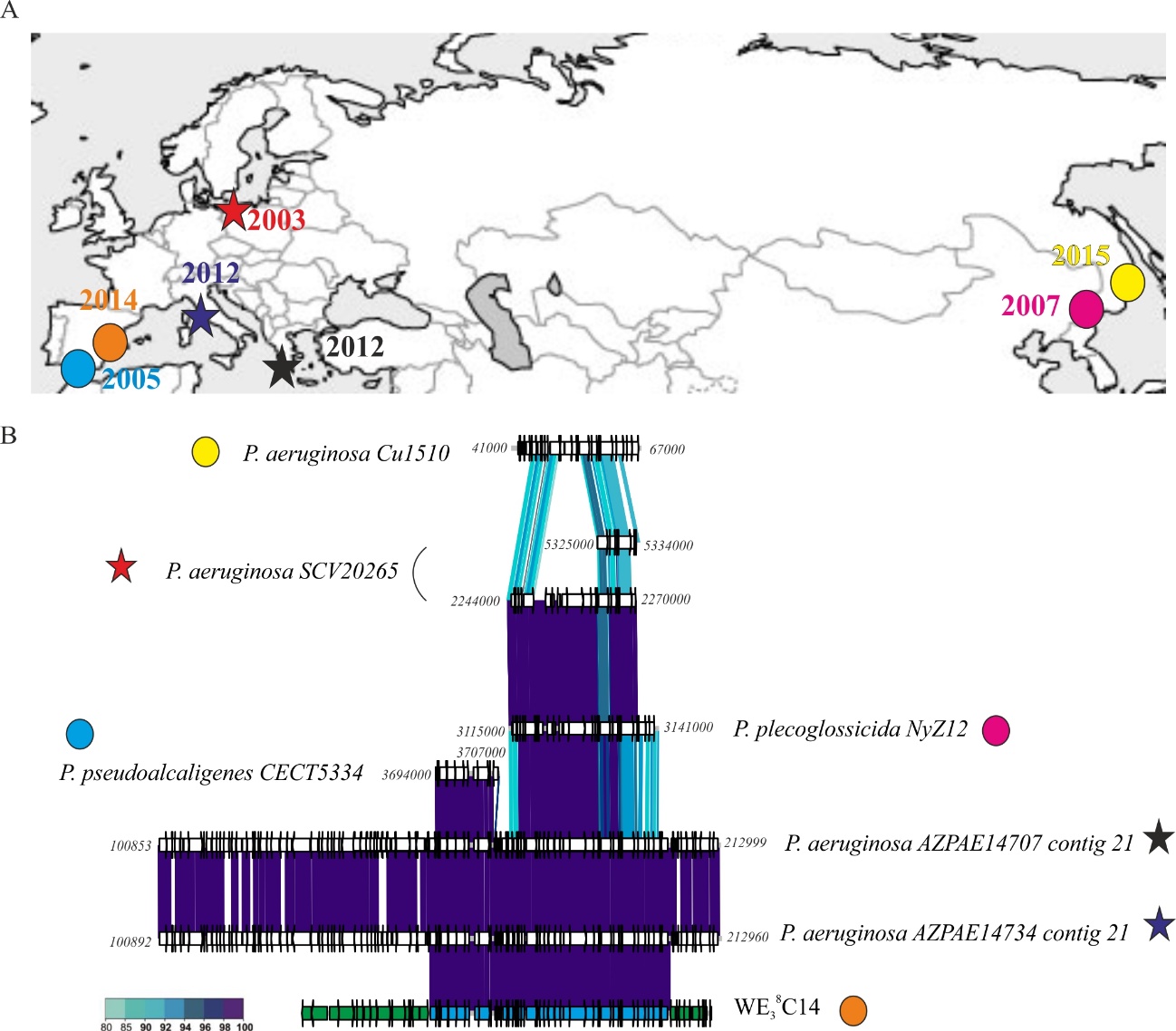


**Figure S17. Distribution of an ICE identified in contigWE_3_^8^C14 between *Pseudomonas* strains.** A) The locations and year of isolation of *Pseudomonas* strains carrying the ICE are marked in the map. Strains isolated from patients are marked with a star and environmental isolates with a circle B) Comparison of ICE in a *Sphingobium* *yanoikuyae* contig to other *Pseudomonas* genomes deposited in NCBI using BLASTN. A minimum nucleotide identity of 80% was used to filter the results. A color legend for BLASTN hits is shown at bottom left.


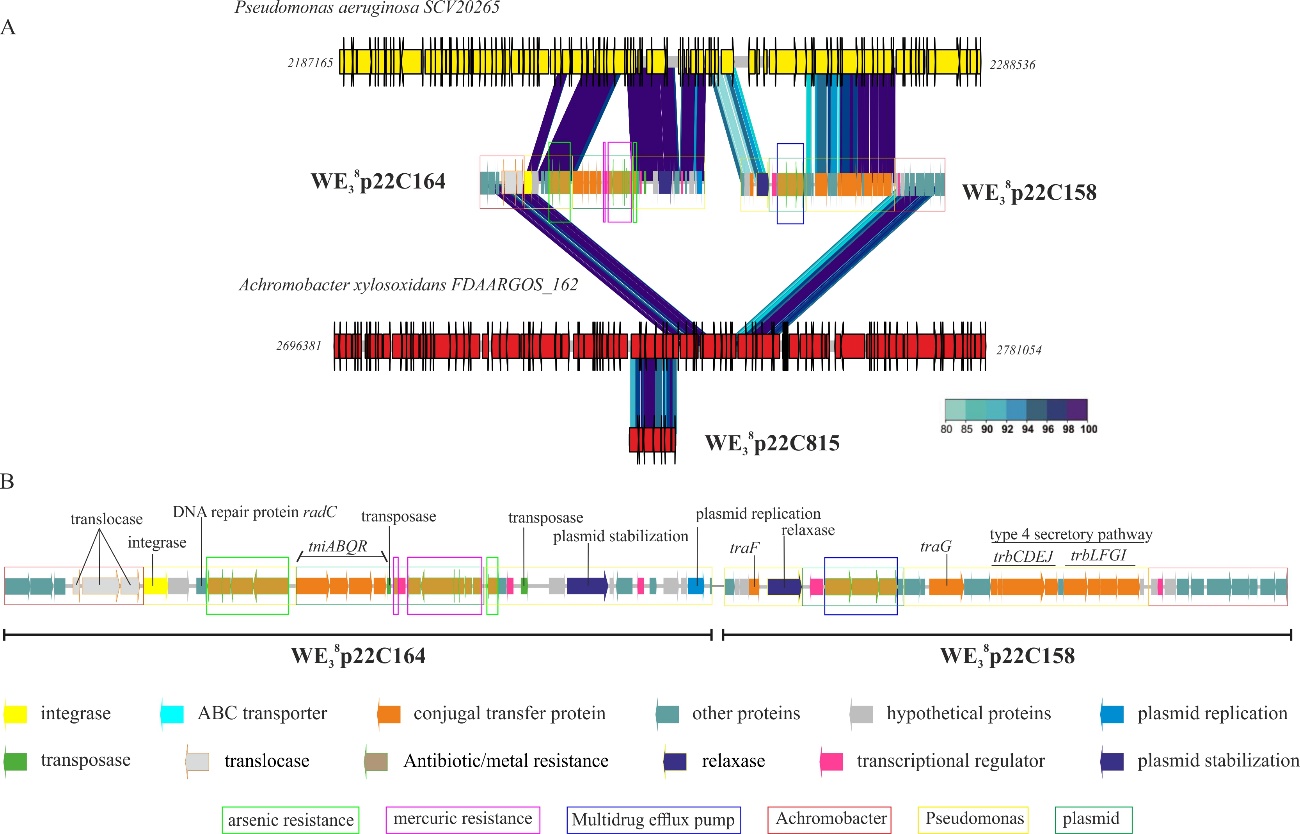


**Figure S18. Exchange of long DNA stretches between genera with similar %GC of the genome.** A) An MGE separated in two contigs compared to an *Achromobacter* and *Pseudomonas* genomes using BLASTN. Minimum identity of 80 was considered for filtering results. B) Annotated view of the MGE shown in A.

1. Ghai R, Hernandez CM, Picazo A, Mizuno CM, Ininbergs K, Díez B, et al. Metagenomes of Mediterranean coastal lagoons. Sci. Rep. [Internet]. 2012 [cited 2012 Oct 4];2:490.
